# Population-specific expression of antimicrobial peptides conferring pathogen resistance in the invasive ladybird *Harmonia axyridis*

**DOI:** 10.1038/s41598-018-21781-4

**Published:** 2018-02-26

**Authors:** Tobias Gegner, Henrike Schmidtberg, Heiko Vogel, Andreas Vilcinskas

**Affiliations:** 1 Institute for Insect Biotechnology, Justus-Liebig-University of Giessen, Heinrich-Buff-Ring 26-32, 35392 Giessen, Germany; 2 Entomology Department, Max-Planck Institute for Chemical Ecology, Hans-Knoell-Strasse 8, 07745 Jena, Germany; 3 Department of Bioresources, Fraunhofer Institute for Molecular Biology and Applied Ecology, Winchester Strasse 2, 35395 Giessen, Germany

## Abstract

The harlequin ladybird *Harmonia axyridis* has emerged as a model species in the context of invasion biology and possesses an expanded repertoire of antimicrobial peptides (AMPs). Here we measured the expression of 22 AMP genes in adult beetles from native and introduced populations, and from a biocontrol population, allowing us to compare populations differing in terms of invasive performance. Strikingly, we observed population-specific expression profiles for particular AMPs. Following an immune challenge, the genes for Coleoptericin1 (Col1), Coleoptericin-likeB and Defensin1 were induced up to several thousand times more strongly in the invasive populations compared to the native and biocontrol populations. To determine the role of Col1 in pathogen resistance, the corresponding gene was silenced by RNA interference (RNAi), causing higher mortality in beetles subsequently infected with the entomopathogen *Pseudomonas entomophila*. The RNAi-triggered susceptibility to this pathogen was reversed by the injection of a synthetic Col1 peptide. We show that a native population is more susceptible to *P*. *entomomophila* infection than an invasive population. This is the first study demonstrating population-specific differences in the immune system of an invasive species and suggests that rapid gene expression changes and a highly adaptive immune system could promote pathogen resistance and thereby invasive performance.

## Introduction

*Harmonia axyridis*, known as the harlequin ladybird, multicoloured ladybird or Asian ladybird beetle, has emerged as a powerful model species for studies of biological invasions^1^. It is native to Central Asia and has been repeatedly introduced over several decades as a biological control agent for aphids and other pest insects. Initially, its range did not expand and there were no apparent negative consequences, but the species later became globally invasive over a short timescale, allowing it to outcompete native ladybirds such as *Adalia bipunctata* in newly colonized areas such as the United Kingdom^2^. A study combining population genetics with approximate Bayesian computation has reconstructed the invasion route on a global scale^3^. The authors found that the eastern and western populations of *H*. *axyridis* in North America originate from two independent introductions of the native Asian beetle, but that only the eastern population served as the source for at least six independent introductions into other continents, including Europe. This study inspired a hypothesis in which features promoting the invasive success of *H*. *axyridis* were acquired in the eastern population of Northern America^3^. Another study has shown that the laboratory mass-rearing of a European population used for biocontrol has resulted in the selection of mutations that are not prevalent in native or invasive beetles, including mutations that increase susceptibility to the entomopathogenic fungus *Beauveria bassiana*^4^.

*H*. *axyridis* has a robust immune system including the constitutively present chemical defense compound harmonine, and an expanded repertoire of antimicrobial peptides (AMPs)^5,6^. Next-generation sequencing of the immunity-related *H*. *axyridis* transcriptome has revealed 49 genes encoding putative AMPs and 10 genes encoding lysozymes^7^. We selected 22 of these genes and performed quantitative real-time PCR (qPCR) to investigate whether the reported differences in pathogen resistance among native, invasive and biocontrol populations of *H*. *axyridis* reflect differences in the innate immune system. We compared two populations originating from the native range (China and South Korea), two introduced populations (Germany and France), and a biocontrol population which has been kept under laboratory conditions for at least 70 generations^4^. Our comparative analysis using untreated and immune-induced beetles revealed that specific genes, especially Col1, are induced by a bacterial challenge much more strongly in invasive populations than in native or biocontrol populations, suggesting a role in survival after pathogen exposure. A comparative analysis of currently available insect genomes and transcriptomes reveals that coleoptericins and coleoptericin-like AMPs are specific to beetles (Coleoptera)^8^. To investigate the role of Col1 in pathogen resistance, we silenced its expression by RNA interference (RNAi) and compared the mortality of RNAi-treated and untreated beetles infected with the bacterial entomopathogen *Pseudomonas entomophila*. We then injected the RNAi-treated beetles with a synthetic Col1 peptide to see whether this increased resistance to the pathogen. Finally, we compared the mortality of non-invasive beetles from China with invasive beetles from Germany and observed that the invasive population is less susceptible to infection with *P*. *entomophila* than the non-invasive population. The comparative analysis of populations differing in their invasive characteristics should facilitate the identification of features that are responsible for invasive success.

## Materials and Methods

### Collection sites and rearing of *H*. *axyridis*

Adult *H*. *axyridis* beetles representing five populations collected from different geographical sites were used in our experiments. We investigated two native range populations, two invasive populations, and one biocontrol strain. The first invasive population was collected in and around Giessen/Ober-Mörlen, Germany. The second invasive population was collected by the Centre de Biologie pour la Gestion des Populations (CBGP, Montpellier, France) from the Department of Gard. We were also provided with one native population from China and one biocontrol strain reared for many generations under laboratory conditions at the CBGP. We also received specimens of a second native population from South Korea, collected by Seung-Joon Ahn. All ladybird populations were reared in cages at 21 °C, 60% relative humidity, and with a 16 h photoperiod. Bean plants (*Phaseolus vulgaris*) infested with pea aphids (*Acyrthosiphon pisum*) were provided *ad libitum* as a food source. For experimental setups all beetles were kept individually in Petri dishes and were supplied with water and sterile freeze-killed eggs of the grain moth *Sitotroga cerealella* (Katz Biotech AG, Germany).

### AMP gene expression analysis

In order to permit comparability of this study with related studies from other insects, we used a mixture of suspended *Micrococcus luteus* DSM 20030 (Gram-positive) and *Escherichia coli* D31 (Gram-negative) to elicit strong immune responses in the beetles and to determine differences in AMP gene expressions between the studied populations, were used to trigger strong immune responses in the beetles. We injected 4 µl of a mixture of heat-inactivated bacteria, which was diluted in phosphate buffered saline (PBS) to 4.75 × 10^8^ cfu. The suspension was injected at the third coxal base into the hemocoel of each adult beetle using a Nanolitre 2000 microinjector and a Sy-Micro4 controller (World Precision Instruments). Non-injected *H*. *axyridis* were used as controls and the beetles were frozen in liquid nitrogen 24 h post-injection and stored at −80 °C. For quantitative real-time PCR (qPCR) six beetles (3 males and 3 females) for each treatment were pooled. The experiment was carried out five times per population.

### RNA isolation, cDNA synthesis and qPCR

Total RNA was isolated with TRI-Reagent® and the Direct-zol™ RNA MiniPrep kit (Zymo Research) according to the manufacturer’s instructions. Pooled samples were ground in liquid nitrogen and RNA purification, including the in-column treatment with 30 U DNase I (Zymo Research), was carried out using 50 mg of homogenized insect tissue. RNA purity and quantity were determined using a Nanodrop ND-1000 spectrophotometer (Thermo Fisher Scientific). We prepared cDNA using the First Strand cDNA Synthesis kit (Thermo Fisher Scientific) by incubating 500 ng total RNA with a 3:1 mix of random and oligo-dT_18_ primers at 65 °C for 5 min, followed by reverse transcription at 25 °C for 5 min, and at 37 °C for 60 min, before terminating the reaction at 70 °C for 5 min. QPCR was performed on a Stratagene Mx3000P system (Biorad) with the SensiMix™ SYBR® No-Rox kit (Bioline) according to the manufacturers’ protocols. We used 10-μl reactions comprising 5 μl 2 × SensiMix™, 1 μl 1:10 diluted cDNA (~50 ng RNA) and 250 nM of each primer, which were designed as previously described^7^ (Supplementary Information, Table S1). Twenty-two AMP genes were selected based on the distinguishability of closely related gene family members, on the clear discrimination of primer pairs, and on the completeness of existing gene sequences. The selected AMP spectrum encompasses member of all identified AMP families. Two technical replicates were run per sample and the results were averaged. The temperature profile consisted of an initial activation at 95 °C for 15 min, followed by 40 cycles of denaturation at 95 °C for 15 s, annealing at 55 °C for 15 s, and extension at 72 °C for 15 s. Specific amplification was verified by subsequent dissociation curve analysis with 0.5 °C increments from 65 °C to 95 °C. Relative gene expression levels were calculated by the ΔΔCT method^9^. Normalization was done with the ribosomal protein gene *RPS3*.

### RNAi-mediated silencing of Col1

Double stranded RNA (dsRNA) for Col1 was prepared using the Ambion MEGAscript T7 kit (Applied Biosystems) according to the manufacturer’s protocol. A 320-bp template for dsRNA was generated by PCR with gene-specific primers (Supplementary information, Figure S1) including the T7 polymerase promoter sequence at the 5′ end (forward: 5′-TAA TAC GAC TCA CTA TAG GGA GTT GCC TGC ATC TCC TTC CAA T-3′, and reverse: 5′-TAA TAC GAC TCA CTA TAG GGA GTT AGC TTT GCC TGG TCC TCT G-3′). Adult *H*. *axyridis* beetles from the invasive population sourced in Germany were injected with 1 µg of Col1 dsRNA (250 nl total volume). Controls were injected with the same volume of water. After 2 days, beetles were infected with 4 µl of a mixture of live bacteria containing 1.7 × 10^9^ cfu/ml *E*. *coli* and 1.1 × 10^9^ cfu/ml *M*. *luteus*. The experiment was carried out four times and five immune-challenged Col1-RNAi specimens, water controls and untreated controls per replicate were pooled and analysed by qPCR at 24 h post-infection (hpi).

### Survival studies

A preliminary test with water-injected beetles infected with different concentrations (8 × 10^3^ cfu/ml, 8 × 10^5^ cfu/ml, 8 × 10^7^ cfu/ml, 8 × 10^9^ cfu/ml) of *P*. *entomophila* and with PBS as a control was conducted to assess the optimal bacterial dosage of 8 × 10^7^ cfu/ml for the further injections (Supplementary Information, Figure S2). The survival experiment with Col1-RNAi beetles (see above) involved two consecutive steps: 1 µg dsRNA (or water as a control) was injected into adult beetles as described above, and after 2 days the beetles were injected with 4 µl of a bacterial suspension comprising 8 × 10^7^ cfu/ml *P*. *entomophila* (or PBS as a control). Ten beetles were used for each treatment (Col1-Pe, Col1-PBS, H_2_O-Pe, and H_2_O-PBS) and the experiment was carried out three times. Individual survival was recorded every day until 10 days post-infection (dpi).

The rescue experiment was carried out as described above for the survival experiment with the addition of a third injection with the synthetic Col1 peptide (BR021). The was synthesized by GenScript based on the natural Col1 peptide sequence^7^ with >90% purity, and was dissolved in 1% (v/v) dimethylformamide (Sigma-Aldrich) in PBS to a concentration of 10 µg/µl. Following the injection of bacteria (see above), the beetles were left for 1.5 hours before we injected 250 nl of Col1 at a concentration of 5 µg/µl in PBS (1.25 µg per beetle). Controls were injected with an equal volume of PBS. Ten beetles were used for each treatment (Col1-Pe-PBS, Col1-Pe-BR021, and H_2_O-Pe-PBS) and the experiment was carried out three times. Individual survival was recorded every day until 10 dpi.

The survival experiment comparing the susceptibility to *P*. *entomophila* of the native population from China and the invasive population from the German population was performed as described before. Ten beetles were used for each treatment (Chi-Pe, Chi-PBS, Ger-Pe, and Ger-PBS) and the experiment was carried out three times. Individual survival was recorded every day until 10 dpi.

### Statistical analysis

Statistical analysis of qPCR data for the AMP gene expression experiment was carried out using R v3.3.3^10^ and the packages multcomp v1.4-0^11^ and sandwich v2.3-3^12^. For each of the AMP genes, a one-way analysis of variance (ANOVA) was used as fitted model with ΔΔCT as the response variable and population as the factor. A parametric multiple comparison analysis was used in the context of each gene by applying general linear hypotheses on the fitted model to test for pairwise differences. Population comparisons were defined as contrasts and p-values were adjusted for multiple testing using the heteroscedasticity robust sandwich estimator for the covariance matrix to calculate simultaneous confidence intervals^12,13^. Normality assumption was verified comparing t-quantile-quantile plots for the standardized residuals of each ANOVA model with t-quantile-quantile plots for 11 simulated, truly t-distributed data sets with equal sample size (Supplementary Information, Figure S3) and the assumption appeared fulfilled for each analysed AMP gene. Significant differences were classified at three levels: p < 0.05, p < 0.01 and p < 0.001. The corresponding R script is available in the supplementary information (File S1).

QPCR data for the RNAi-mediated silencing of Col1 was analysed by a one-way ANOVA with ΔΔCT as the response variable and treatment as the factor.

Survival analysis was carried out using the R package survival v2.40-1^14^. Kaplan-Meier survival curves were plotted and differences in survival rates between treatments were calculated using the log-rank test and Holm-corrected p-values.

## Results

### AMP and lysozyme expression in *H*. *axyridis* beetles

**Figure 1:**
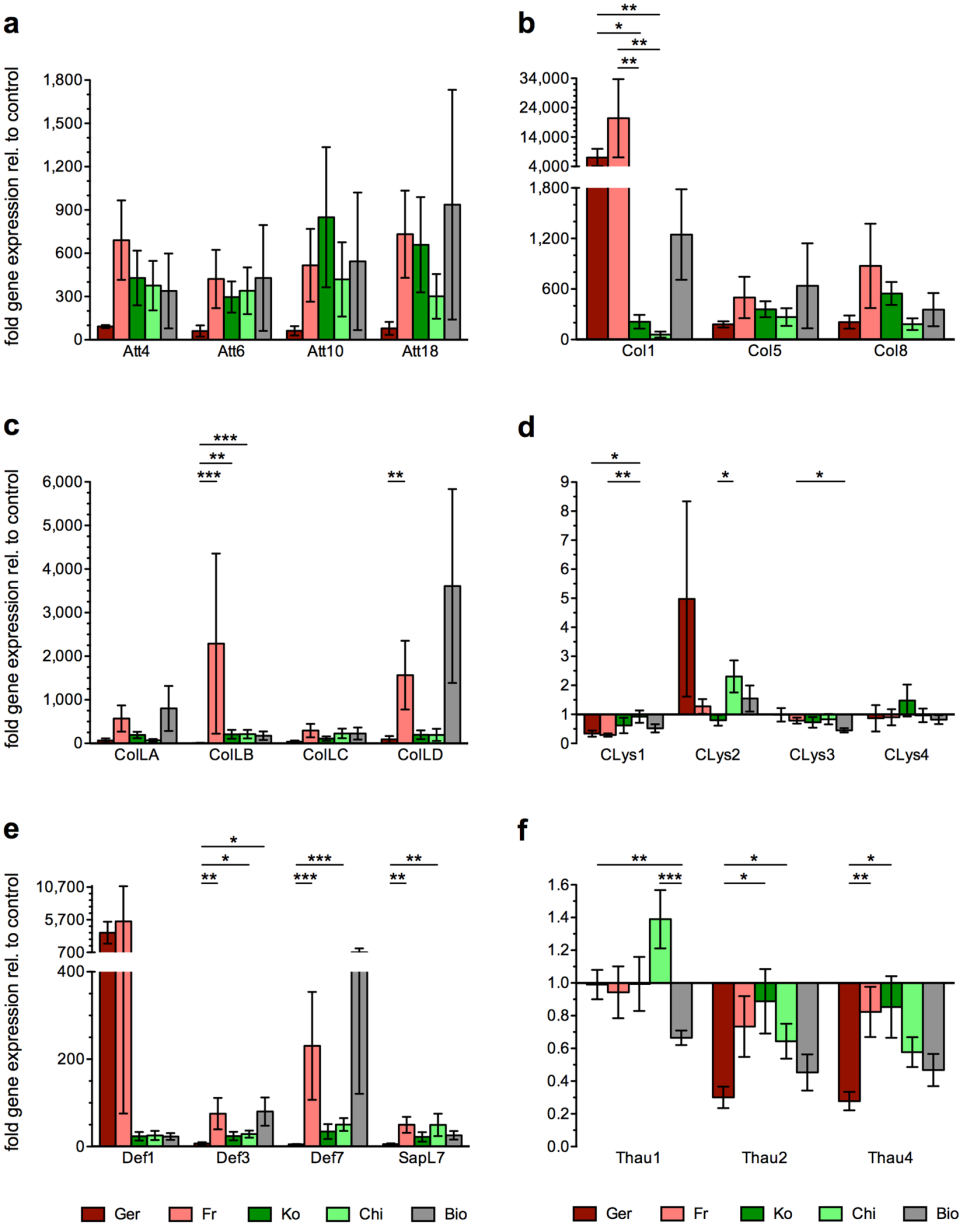
Relative levels of AMP gene expression in challenged beetles (normalized to controls) representing two invasive populations from Germany (Ger, dark red) and France (Fr, light red), two native populations from South Korea (Ko, dark green) and China (Chi, light green), and one biocontrol population (Bio, grey). The expression levels were measured 24 h after beetles were injected with a mixture of heat-inactivated bacteria. Gene families: (**a**) attacins, (**b**) coleoptericins, (**c**) coleoptericin-like peptides, (**d**) c-type lysozymes, (**e**) defensins, and (**f**) thaumatins. Bars show mean values of five biological replicates. Error bars indicate standard errors of the mean. X-axis at control level = 1. Significant differences between two groups are depicted using asterisks and lines. Significance levels: p < 0.05 (*), p < 0.01 (**), p < 0.001 (***).

The expression of genes encoding attacins was upregulated following an immune challenge in four of the populations we tested, with beetles from Germany showing the weakest upregulation (Fig. 1a, Supplementary File S2). Surprisingly, the expression of genes encoding thaumatin family antifungal peptides was downregulated in all five challenged populations compared to untreated controls (Fig. 1f), except for Thaumatin1 in the Chinese population. The most obvious differences among the invasive populations from Germany and France, the native populations from China and South Korea, and the biocontrol population, were observed when we compared the expression levels of genes encoding Col1, Coleoptericin-likeB, Defensin1 and c-type Lysozyme2, which tended to be induced more strongly in the invasive populations. Remarkably, the gene encoding Col1 was upregulated more than 7,000-fold in the invasive population from Germany and more than 20,000-fold in the invasive population from France following the immune challenge, compared to untreated controls (Fig. 1b, Supplementary File S2).

### RNAi-mediated silencing of Col1 and survival of beetles infected with *P*. *entomophila*

**Figure 2:**
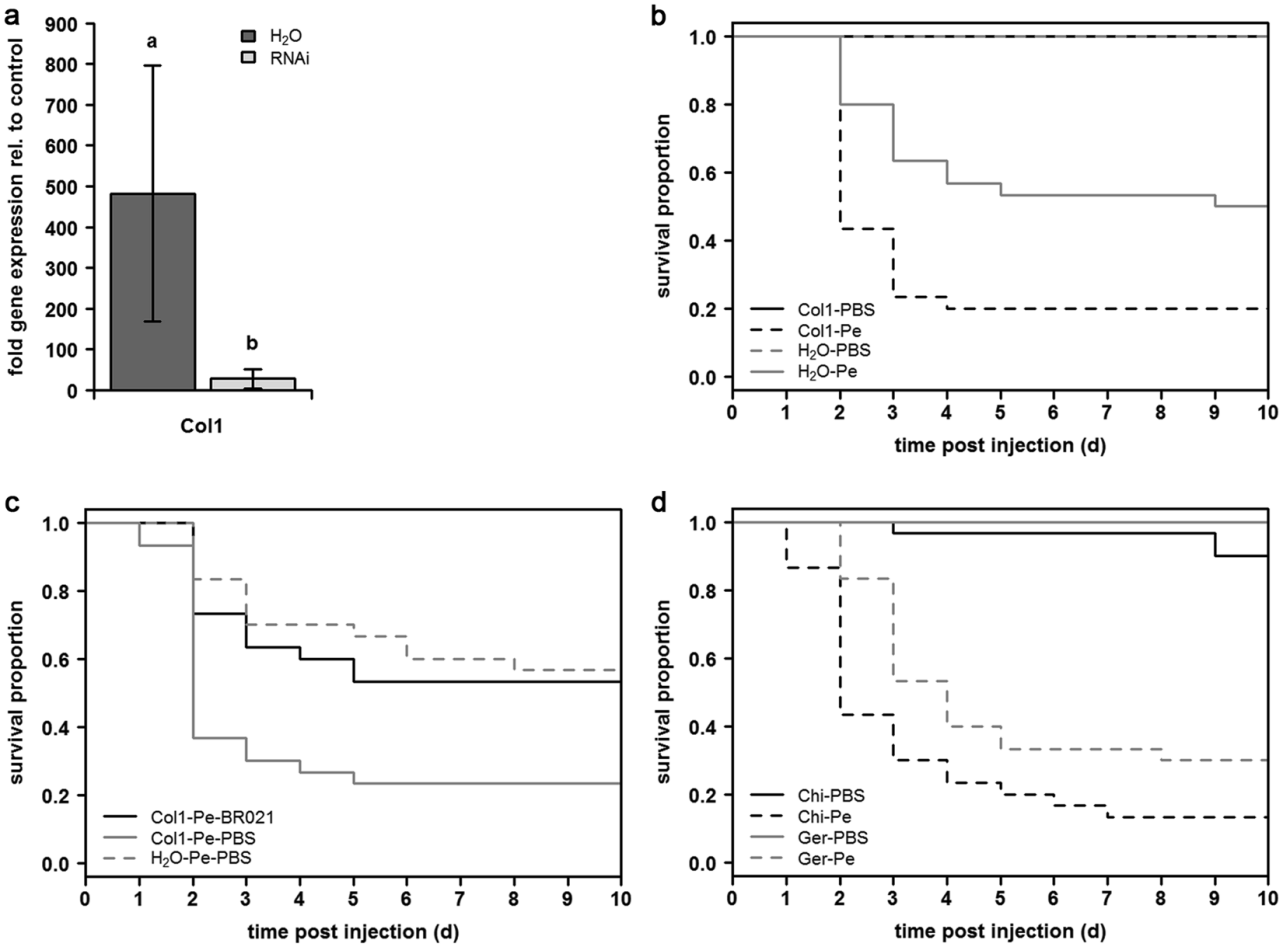
RNAi-mediated knockdown of Col1, the mortality of Col1-RNAi beetles sourced from the invasive German population, and the mortality of *P*. *entomophila* (Pe)-infected native beetles from China compared with Pe-infected invasive German beetles. (**a**) Relative immunity-related gene expression levels in Col1-RNAi beetles compared to water controls (normalized to untreated controls) at 24 hpi. Bars show mean values of four biological replicates with standard deviations. Letters indicate significant differences between treatments (one-way ANOVA: p = 0.0032). (**b**) Kaplan-Meier survival curves of Pe-infected Col1-RNAi and water control beetles and uninfected Col1-RNAi and water control beetles. Lines show mean values of three biological replicates (n = 30 per treatment). Significant differences (log rank test, Holm-corrected p-values): H_2_O-PBS *vs* H_2_O-Pe (p < 0.0001), Col1-PBS *vs* Col1-Pe (p < 0.0001), and H_2_O-Pe *vs* Col1-Pe (p = 0.0076). (**c**) Kaplan-Meier survival curves of Pe-infected Col1-RNAi, Col1-RNAi rescue beetles (with 5 µg/µl synthetic Col1) and water control beetles. Lines show mean values of three biological replicates (n = 30 per treatment). Significant differences (log rank test, Holm-corrected p-values): H_2_O-Pe-PBS *vs* Col1-Pe-PBS (p = 0.0046), Col1-Pe-BR021 *vs* Col1-Pe-PBS (p = 0.0132). (**d**) Kaplan-Meier survival curves of Pe-infected native beetles from China (Chi-Pe) and invasive beetles from Germany (Ger-Pe) and uninfected PBS control beetles (Chi-PBS, Ger-PBS), Lines show mean values of three biological replicates (n = 30 per treatment). Significant differences (log rank test, Holm-corrected p-values): Chi-Pe *vs* Chi-PBS (p < 0.0001), Ger-Pe *vs* Ger-PBS (p < 0.0001), Chi-Pe *vs* Ger-Pe (p = 0.0333).

The injection of invasive *H*. *axyridis* beetles from Germany with dsRNA homologous to the *col1* gene successfully prevented the induction of this gene following the bacterial challenge (Fig. 2a, Supplementary File S3). There was a strong and significant decrease in gene expression relative to unchallenged controls 24 hpi from 482.1 (±314.3 sd) fold in the water-control beetles to 28.2 (±23.9 sd) fold in the RNAi-treated beetles (p = 0.0032). Challenge of adult *H*. *axyridis* beetles with the entomopathogenic bacterium *P*. *entomophila* resulted in high mortality rates in a dose-dependent fashion (Supplementary Information, Figure S2). The knockdown of *col1* further reduced the survival of beetles challenged with *P*. *entomophila*, with the mortality rate increasing from 50% in the water-control beetles to 80% in the RNAi-treated beetles at 10 dpi (p = 0.0076) (Fig. 2b, Supplementary File S4).

### Reinstatement of pathogen resistance by a synthetic Col1 peptide

The importance of Col1 for the ability of *H*. *axyridis* to resist *P*. *entomophila* was confirmed by injecting the Col1-RNAi beetles with 1.25 µg of the synthetic Col1 peptide BR021 (Fig. 2c, Supplementary File S4). There was a significant decrease in mortality from 77% in the Col1-Pe-PBS group to 47% in the Col1-Pe-BR021 group (p = 0.0132), which was comparable to the 43% mortality in the H_2_O-Pe-PBS control beetles (p = 0.6764). The mortality levels in the control groups in this experiment were similar to those observed in the RNAi experiment above, i.e. 50%/43% mortality in the negative controls and 80%/77% mortality in the RNAi group without a subsequent BR021 injection. These data confirmed that the third injection *per se* has no further negative effect on survival, and the reinstatement of resistance can therefore be assigned completely to the effect of the injected peptide. As in the previous experiment, the difference in mortality between uninfected RNAi-treated beetles and controls was significant (p = 0.0046 for H_2_O-Pe-PBS *vs* Col1-Pe-PBS).

### Higher susceptibility to *P*. *entomophila* infection in the non-invasive population

We compared the mortality of non-invasive beetles from China with invasive beetles from Germany, to assess if the invasive population is more resistant to infection with *P*. *entomophila* than the non-invasive population (Fig. 2d, Supplementary File S4). Both populations showed a high mortality rate at 10 dpi, which significantly differed from control beetles. Around 87% of infected native beetles and 70% of infected invasive beetles died compared to 10% uninfected native and 0% uninfected invasive beetles (Chi-Pe *vs* Chi-PBS: p < 0.0001, Ger-Pe *vs* Ger-PBS: p < 0.0001). There was no difference in mortality of controls between populations, but the mortality of infected individuals was significantly higher in native beetles than in invasive beetles (p = 0.0333). We further found that the non-invasive population reached the median survival time (ST_50_) after two days and therefore succumbed faster to *P*. *entomophila* infection than the invasive population, which reached the ST_50_ after day 3.

## Discussion

A recent comparative analysis of the immunity-related transcriptomes of three ladybird beetles revealed an expansion in the number of AMPs in the invasive ladybird *H*. *axyridis* compared to the native ladybirds *C*. *septempunctata* and *A*. *bipunctata*^15^. The gradient of AMP diversity and induction ratio from *A*. *bipunctata* to *H*. *axyridis* corresponds to a gradient of increasing resistance against microsporidia and the entomopathogenic fungus *B*. *bassiana*^15–18^.

Here, we compared the induction of 22 genes encoding selected AMPs and lysozymes following the injection of bacteria into *H*. *axyridis* beetles originating from two native populations, two invasive populations from introduced areas, and one biocontrol population. These five populations exhibit differences in their invasive capabilities, with the introduced populations showing the greatest invasive success. The two European populations, originating from the eastern population of North America that has expanded its global range^3^, were characterized by a much higher induction ratio for certain AMP genes compared to the native beetles and those from the biocontrol strain. The latter is more susceptible to *B*. *bassiana* infection than both native and invasive populations, and this has been interpreted as an adverse genetic change resulting from captive breeding^4^.

The experimental activation of immune responses in beetles of *H*. *axyridis* elucidated that invasive populations display remarkably higher induction levels of particular AMPs such as Col1 and Def1 when compared with those from native populations. Our data indicate that population-specific AMP gene expression and the induction levels of individual AMPs are dynamic, and may change more rapidly than previously thought. The simultaneous induction of multiple AMP genes can synergistically enhance pathogen resistance due to the ability of some AMPs to potentiate the activity of other AMPs^19^. Indeed, Col1 has been shown to display synergistic activity with c-type lysozyme in *H*. *axyridis*^20^. To the best of our knowledge, the current study is the first demonstrating population-specific differences in the immune system of an invasive species and suggests that rapid gene expression changes and a highly adaptive immune system could promote pathogen resistance and thereby invasive performance. To confirm this hypothesis, we tested the ability of invasive beetles to resist the entomopathogen *P*. *entomophila* before and after silencing the gene encoding Col1, and subsequently following the administration of a synthetic Col1 peptide. We found that the mortality of the invasive beetles increased significantly from 50% to 80% following the RNAi-mediated knockdown of the *col1* gene, but that resistance was completely restored in RNAi-treated beetles which were provided with the synthetic Col1 peptide. The injection of synthetic Col1 significantly decreased the mortality from 77% to 47%, which is comparable with the initial increase in mortality after *col1* gene knockdown. As far as we are aware, this is the first direct confirmation of the protective role of a specific AMP in a non-model insect both by silencing its expression to increase mortality following a bacterial challenge, and by restoring full resistance by injecting a synthetic analogue of the peptide into beetles affected by *col1* silencing. The importance of Col1 for invasive populations is further strengthened by our comparative survival experiment with non-invasive beetles from China and invasive beetles from Germany. We found that the non-invasive population was more prone to infection with *P*. *entomophila* than the invasive population, i.e. a faster death rate and a higher mortality of 87% of the native beetles in comparison to 70% mortality of the invasive beetles. We propose that the observed differences in Col1 gene expression translate into higher pathogen resistance of invasive populations.

Our study demonstrates that invasive and non-invasive populations are distinguished by their expression of several immune genes, particularly Col1, and that Col1 is an important component of the immune repertoire of an invasive population. These findings potentially support the hypothesis that immunology plays a role in biological invasions^21,22^ and expands this assumption from the species level to the population level. It is widely accepted that bottlenecks in population size can reduce genetic diversity and increase inbreeding, causing inbreeding depression. However, even though comparative analysis has shown that *H*. *axyridis* has indeed endured a bottleneck, this species has purged the alleles causing inbreeding depression in the invasive populations^23^. Furthermore, a rapid increase in the dispersal of *H*. *axyridis* during range expansion has been documented^24^. Our observations agree with these studies and suggest that the rapid evolution of a novel trait (in this case, the stronger induction of particular AMPs such as Col1) in *H*. *axyridis* populations can potentially contribute to their invasive performance.

### Data accessibility

All data are accessible in the Supplementary Information.

## Electronic supplementary material

Supplementary information

Supplementary File S1

Supplementary File S2

Supplementary File S3

Supplementary File S$
