# Supplementary material for: Population-specific expression of antimicrobial peptides conferring pathogen resistance in the invasive ladybird Harmonia axyridis
Source: Sci Rep. 2018 Feb 26;8:3600. doi: 10.1038/s41598-018-21781-4 (PMC5827023; doi:10.1038/s41598-018-21781-4)

Supplementary information to the manuscript

Population-specific expression of antimicrobial peptides conferring pathogen resistance in the invasive ladybird *Harmonia axyridis*

Tobias Gegner[1], Henrike Schmidtberg[1], Heiko Vogel[2] and Andreas Vilcinskas[1,3,*]

[1] Institute for Insect Biotechnology, Justus-Liebig-University of Giessen, Heinrich-Buff-Ring 26-32, 35392 Giessen, Germany

[2] Entomology Department, Max-Planck Institute for Chemical Ecology, Hans-Knoell-Strasse 8, 07745 Jena, Germany

[3] Department of Bioresources, Fraunhofer Institute for Molecular Biology and Applied Ecology, Winchester Strasse 2, 35395 Giessen, Germany

* Corresponding author, email: Andreas.Vilcinskas@agrar.uni-giessen.de

Supplementary Table and Figures

Table S1. List of 23 gene-specific primer pairs used for AMP gene expression analysis (22 AMPs and RPS3 as housekeeping gene for normalization of qPCR data).

Figure S1. The *col1* gene sequence with the corresponding binding sites for dsRNA and qPCR primers.

Figure S2. Kaplan-Meier survival curves for dose-dependent survival of water-injected beetles after injection with different concentrations (8×10^3 cfu/ml, 8×10^5 cfu/ml, 8×10^7 cfu/ml, 8×10^9 cfu/ml) of *P. entomophila* (Pe) or with PBS as a control (n = 10 per treatment).

Figure S3. Verification of the normality assumption for the AMP gene expression analysis data. Assumption was verified per gene by comparison of the t-quantile-quantile plot for standardized residuals of the ANOVA model (red graph) with t-quantile-quantile plots for 11 simulated, truly t-distributed data sets with equal sample size (black graphs). Genes: a) Attacin4, b) Attacin6, c) Attacin10, d) Attacin18, e) Coleoptericin1, f) Coleoptericin5, g) Coleoptericin8, h) Coleoptericin-likeA, i) Coleoptericin-likeB, j) Coleoptericin-likeC, k) Coleoptericin-likeD, l) C-type Lysozyme1, m) C-type Lysozyme2, n) C-type Lysozyme3, o) C-type Lysozyme4, p) Defensin1, q) Defensin3, r) Defensin7, s) Sapecin-like Defensin7, t) Thaumatin1, u) Thaumatin2, v) Thaumatin4.

Supplementary Files

File S1 R-Script for AMP gene expression analysis

File S2 qPCR data for AMP gene expression analysis

File S3 qPCR data for Col1-RNAi knockdown verification

File S4 Survival data

Table S1

Primer	Target gene	Sequence forward (5’-3’)	Sequence reward (5’-3’)
Att4	Attacin4	CAAGAATCCAGGAGGTACTCAAG	ACCGCCTCCTAATGTTGTTG
Att6	Attacin6	AAGAATCCAGGAGGTACACAGG	ACCGCCTCCTAATGTTGTTG
Att10	Attacin10	CGCTTTAGCCTCTTCAGAATCA	TCCTTTATGCCCCAAAGTCA
Att18	Attacin18	ACCCACAAGGTACGCAGGT	CCTCCTCCTAATGCTGTTGG
Col1	Coleoptericin1	CTGCATCTCCTTCCAATATGC	GGTCCTTCGGGAACAACATA
Col5	Coleoptericin5	CATTTGCCTGCATTTACTTCC	TTGGGGCTCCAGGTAGAAG
Col8	Coleoptericin8	GGAAGAGGTTGTTGTAGATGGAG	TCTGGTGAGTGATGGGTCAA
ColLA	Coleoptericin-likeA	GAAGGATGGCAAGTCGAACA	ACCACGTGAAGGTTCCTCCT
ColLB	Coleoptericin-likeB	AGATGTGCCTTGCTTCGAGA	ATTGTGCGAGAACCACCAAC
ColLC	Coleoptericin-likeC	CATGTTCGGGACTCTGAAGG	CTCACAGATCCGGCAGTGTT
ColLD	Coleoptericin-likeD	CACGTTCGGGATACTGATGG	CACCTGAAGGTTGCTCCAAG
CLys1	C-type Lysozyme1	GCGTTTGTGCAGAGAAGGTC	TAACATGGCGGAAGTGGAAG
CLys2	C-type Lysozyme2	TCAAAGCTTGGAATGGTTGG	TCCATCAACATAGCGGCATT
CLys3	C-type Lysozyme3	GGGAATTCCAGGCAATCAGT	CATGGCAACCTTTACCAGGA
CLys4	C-type Lysozyme4	GGGTCAGAAGCCCGAATTTA	CAGCGGTGTCAAAGTTGGAT
Def1	Defensin1	CATTCTCCTCTCCTACCGAACC	GGCGACACAATGTAATGCAC
Def3	Defensin3	ACCGAAAGGGGAAATTCTTC	GGCGACACAATGTAATGCAC
Def7	Defensin7	TGAGTATCGAAGCAGGAACCA	TCCTCCCTCGTAGTTGAAGG
SapL7	Sapecin-like Defensin7	ACCGAACCGAAAGAGGAAAT	GCAGCTTAAAGCACATGCAG
Thau1	Thaumatin1	TTCTGGGGAAGAAGTTGGTG	CTCGACAAGGGTAGCTGGAG
Thau2	Thaumatin2	TGAAGGTGGACAAGTGTGGA	CAGGGGCATAAACAGAGACC
Thau4	Thaumatin4	AGGGCTGGACTTCTACGACA	TCCTGGACAATGGTTGTTGA
RPS3	Ribosomal proteine RPS3	GGCTACCAGAACCGACAGAG	GTGCTATGGCGCATAATCCT

Figure S1

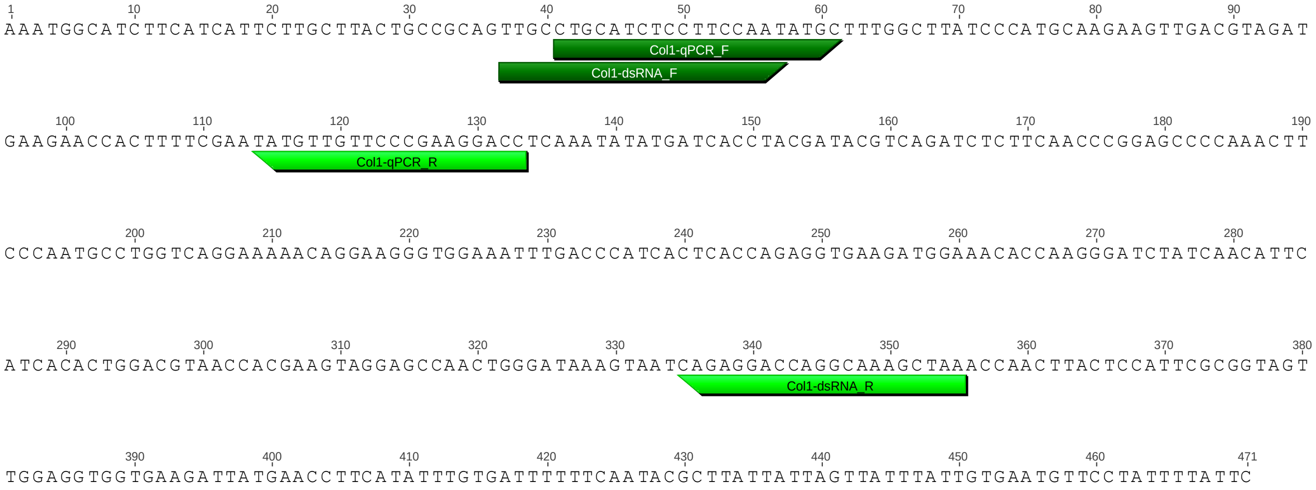

Figure S2

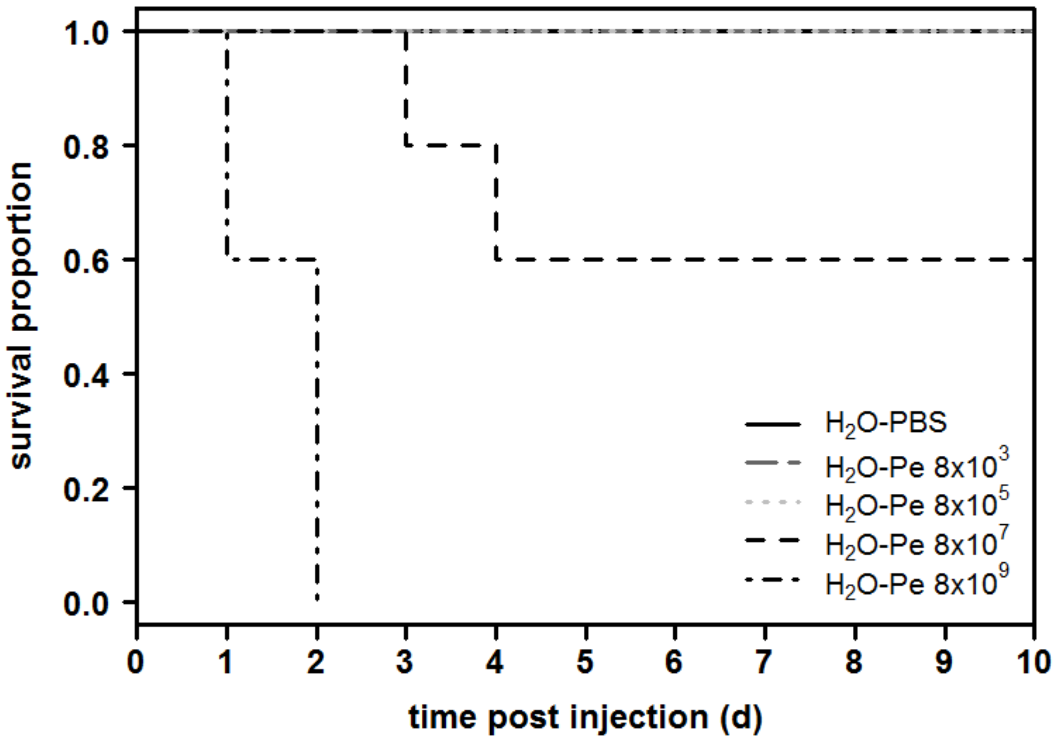

Figure S3 a

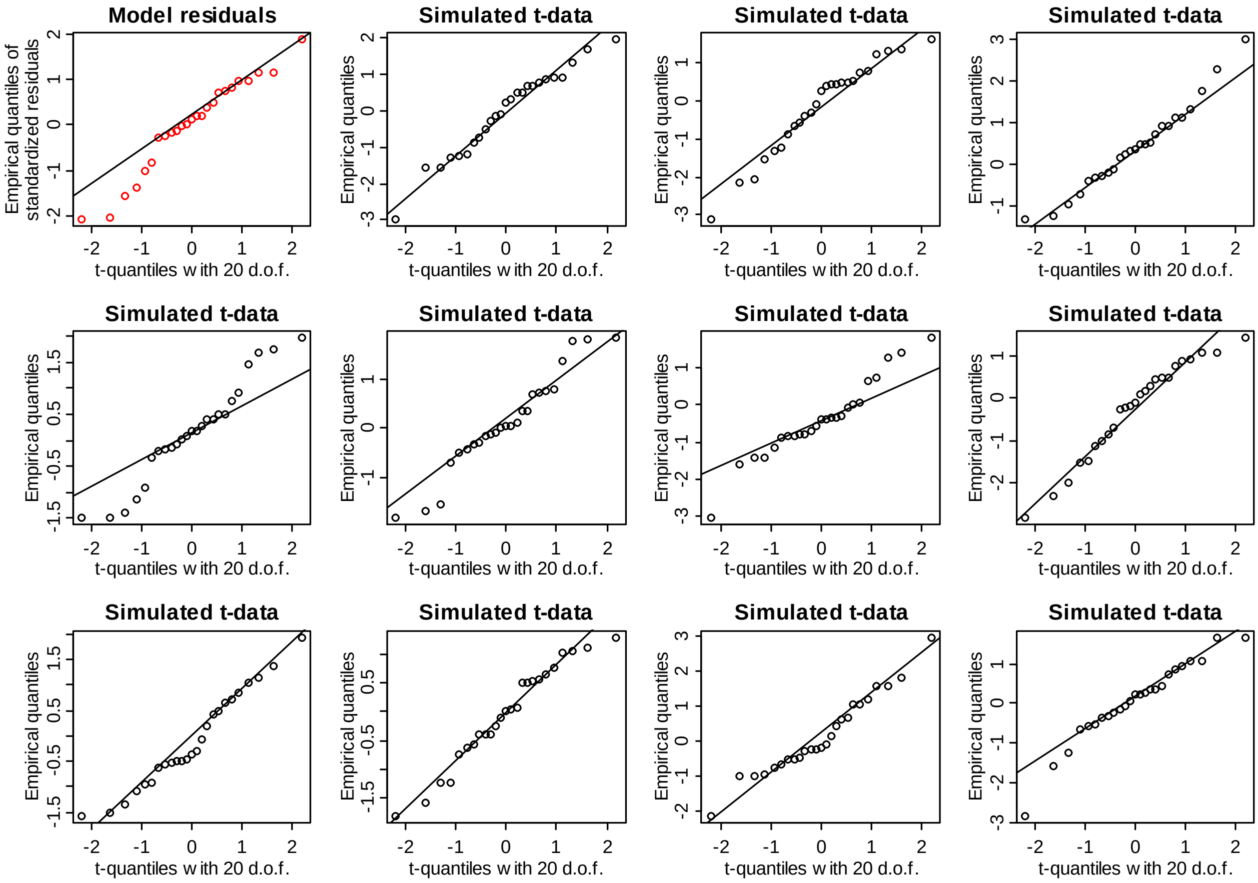

Figure S3 b

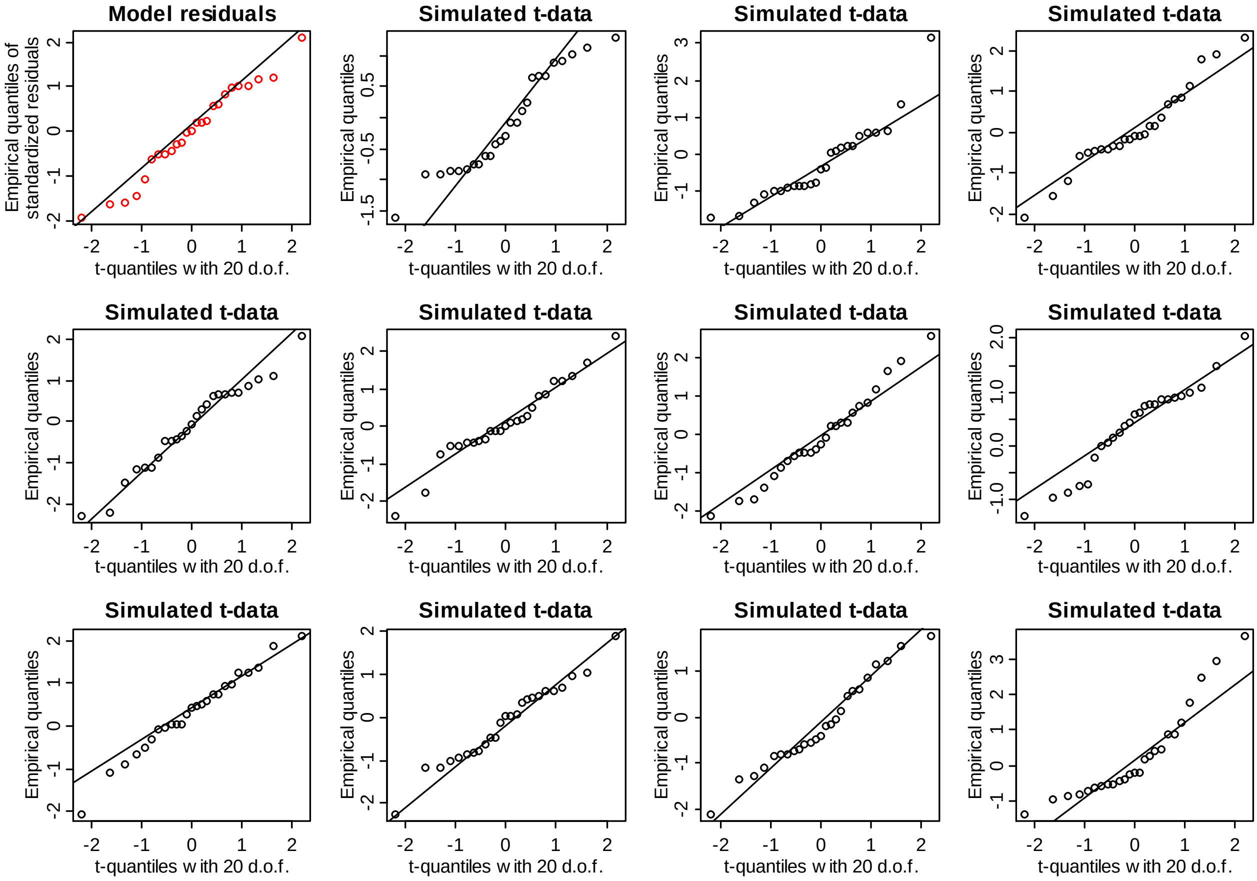

Figure S3 c

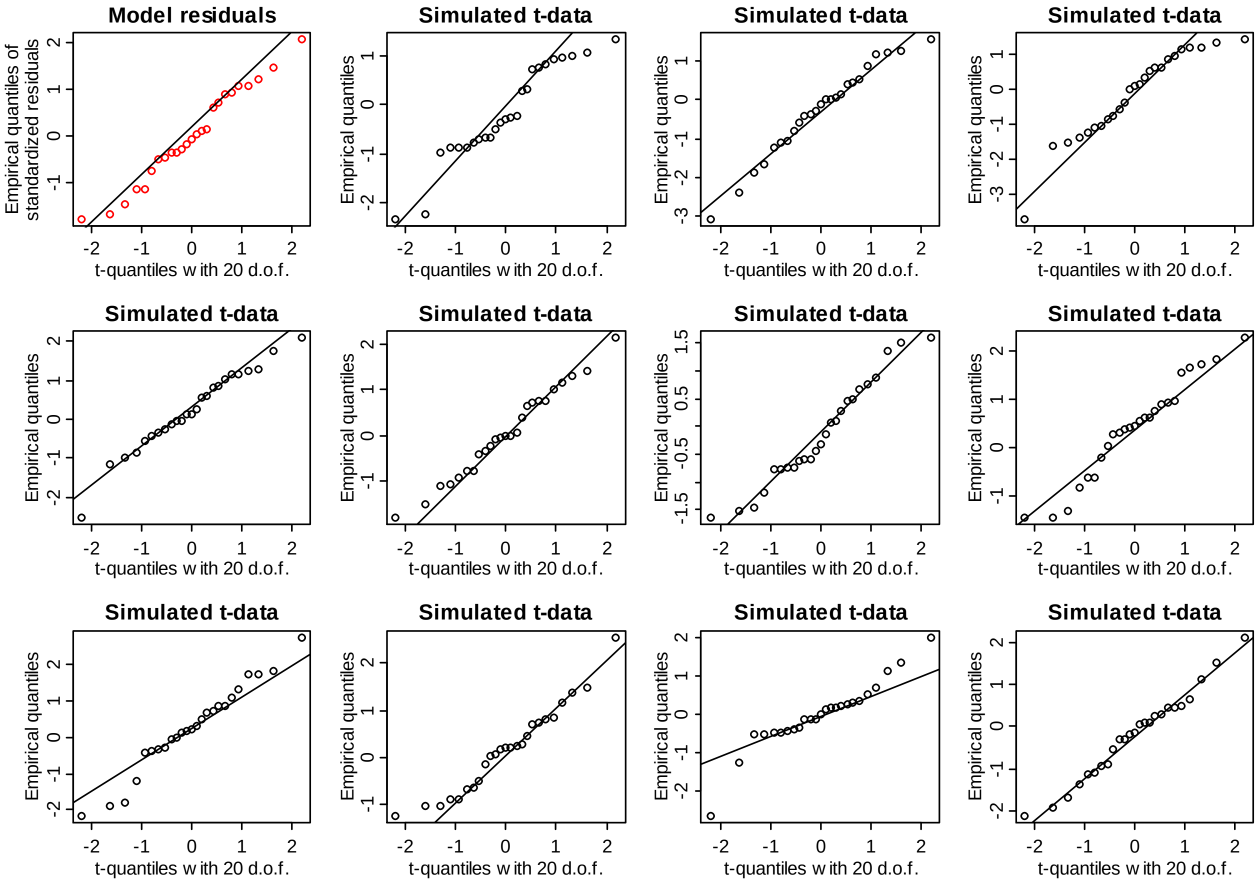

Figure S3 d

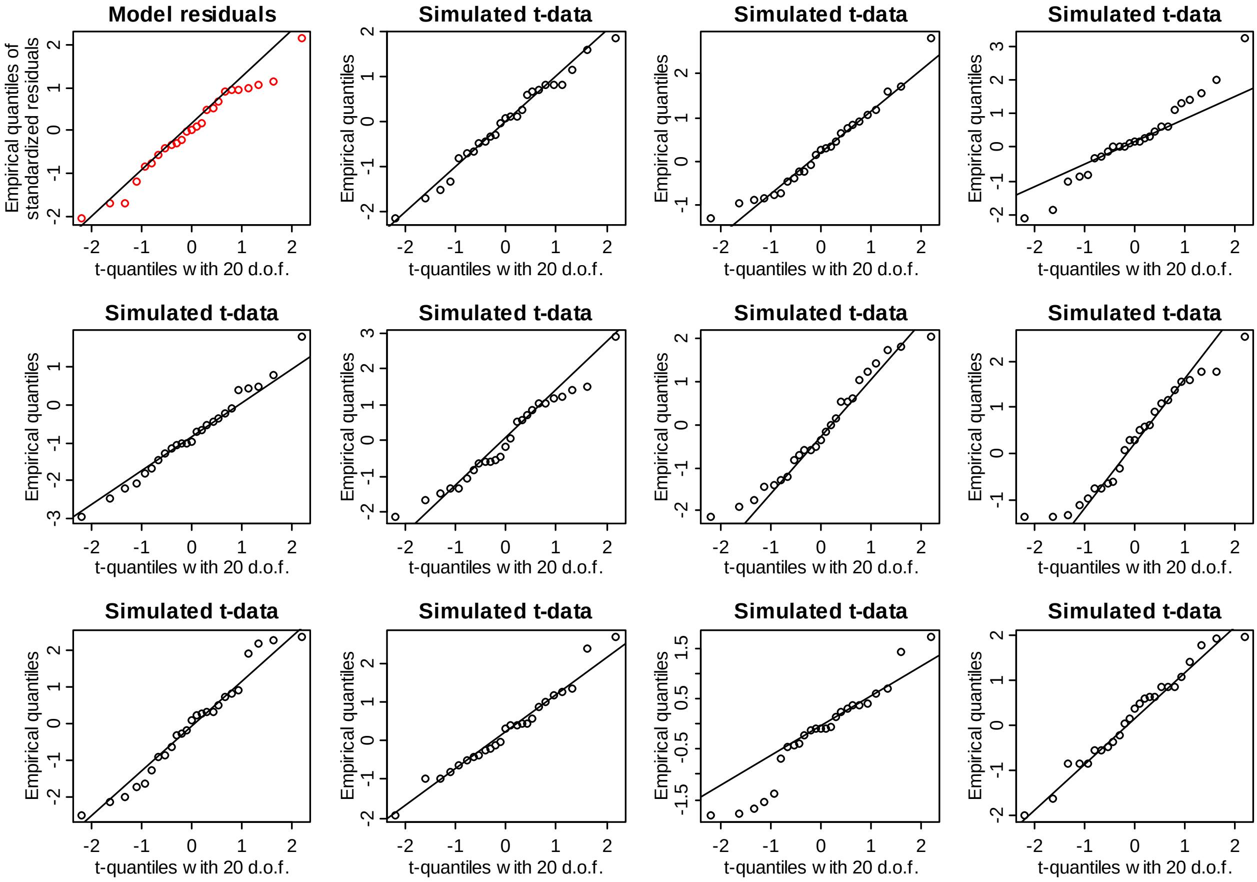

Figure S3 e

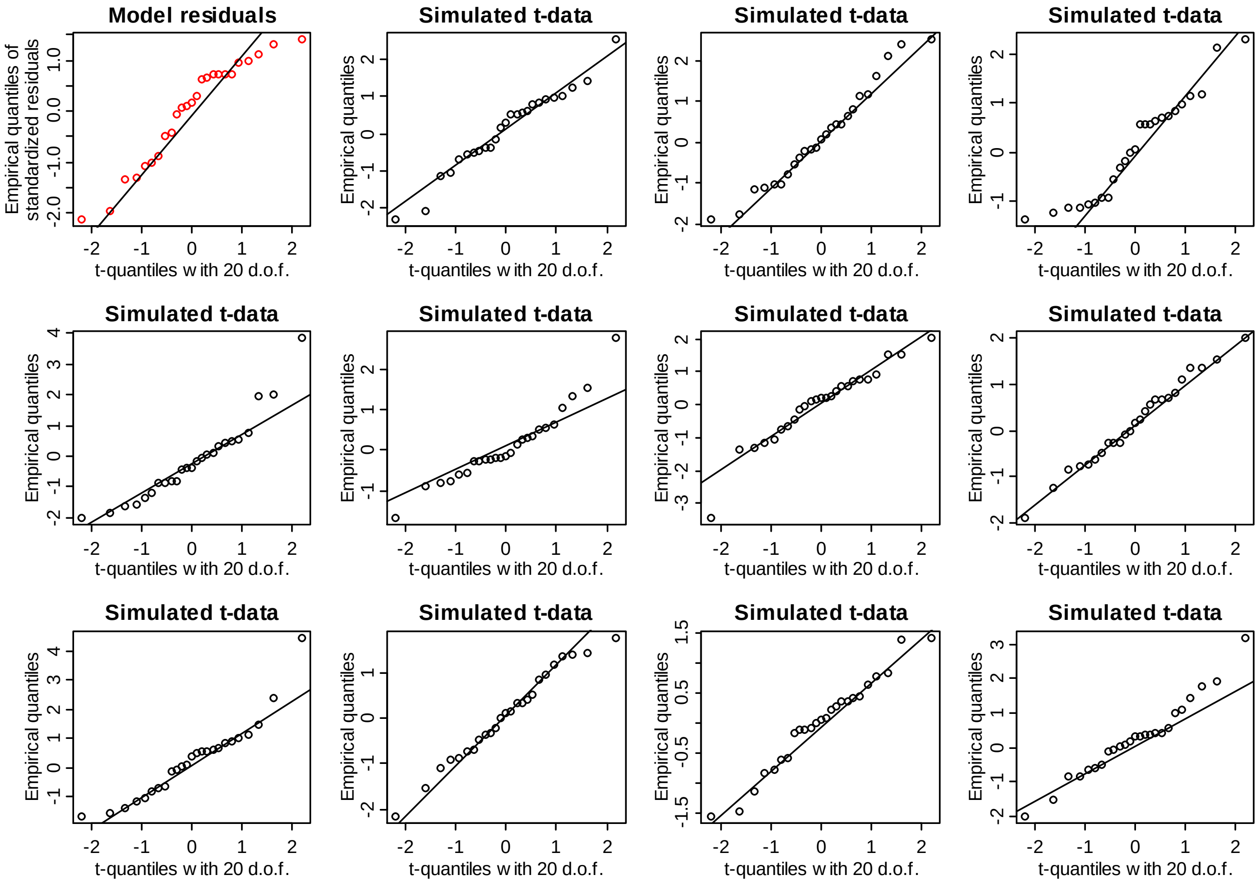

Figure S3 f

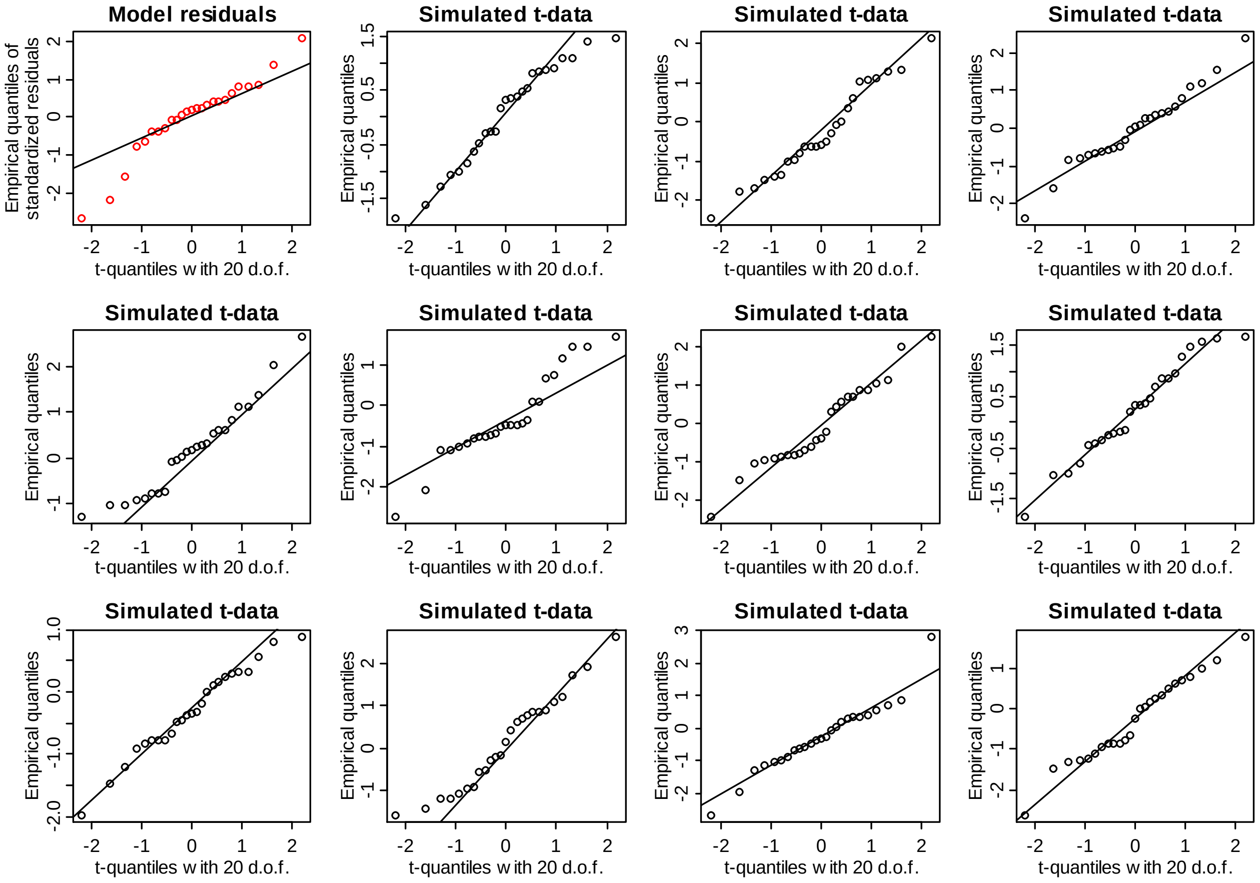

Figure S3 g

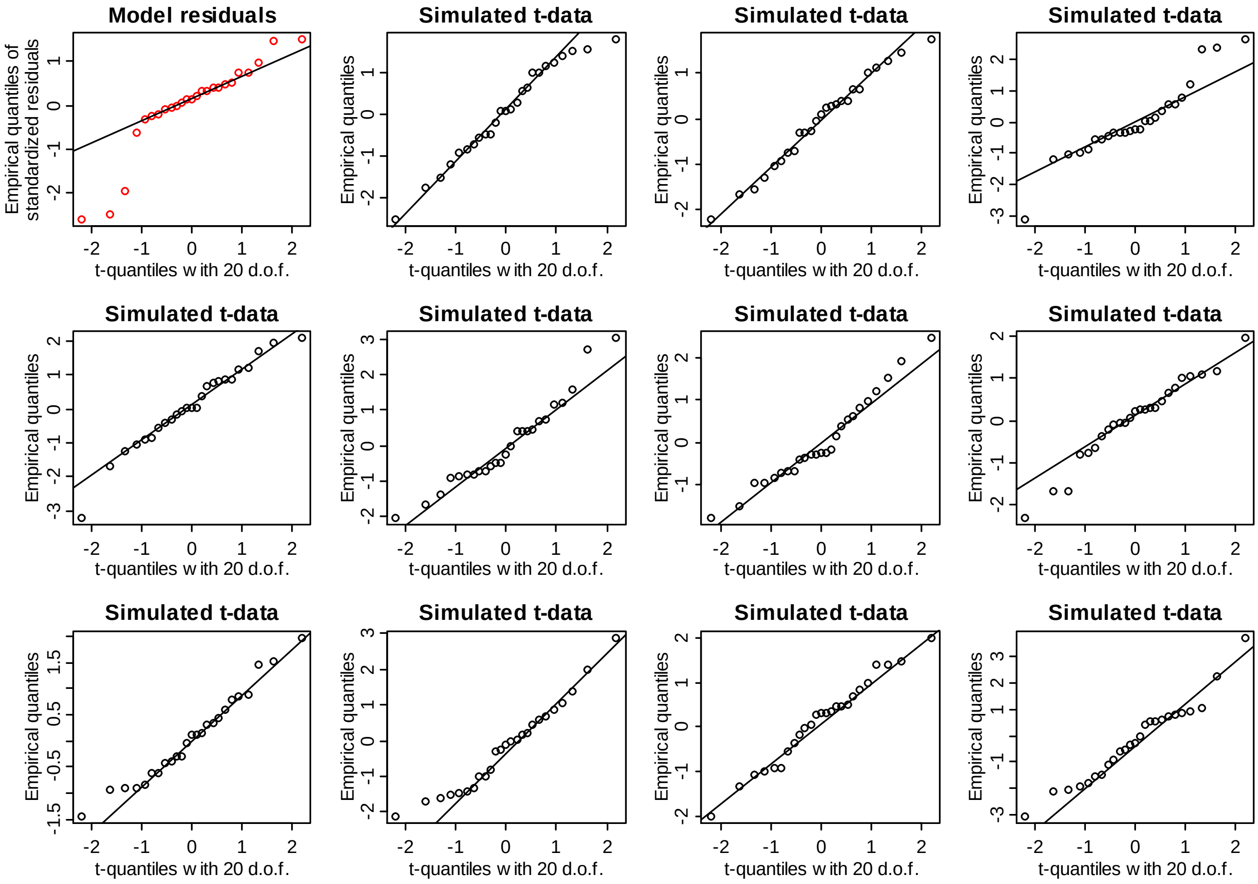

Figure S3 h

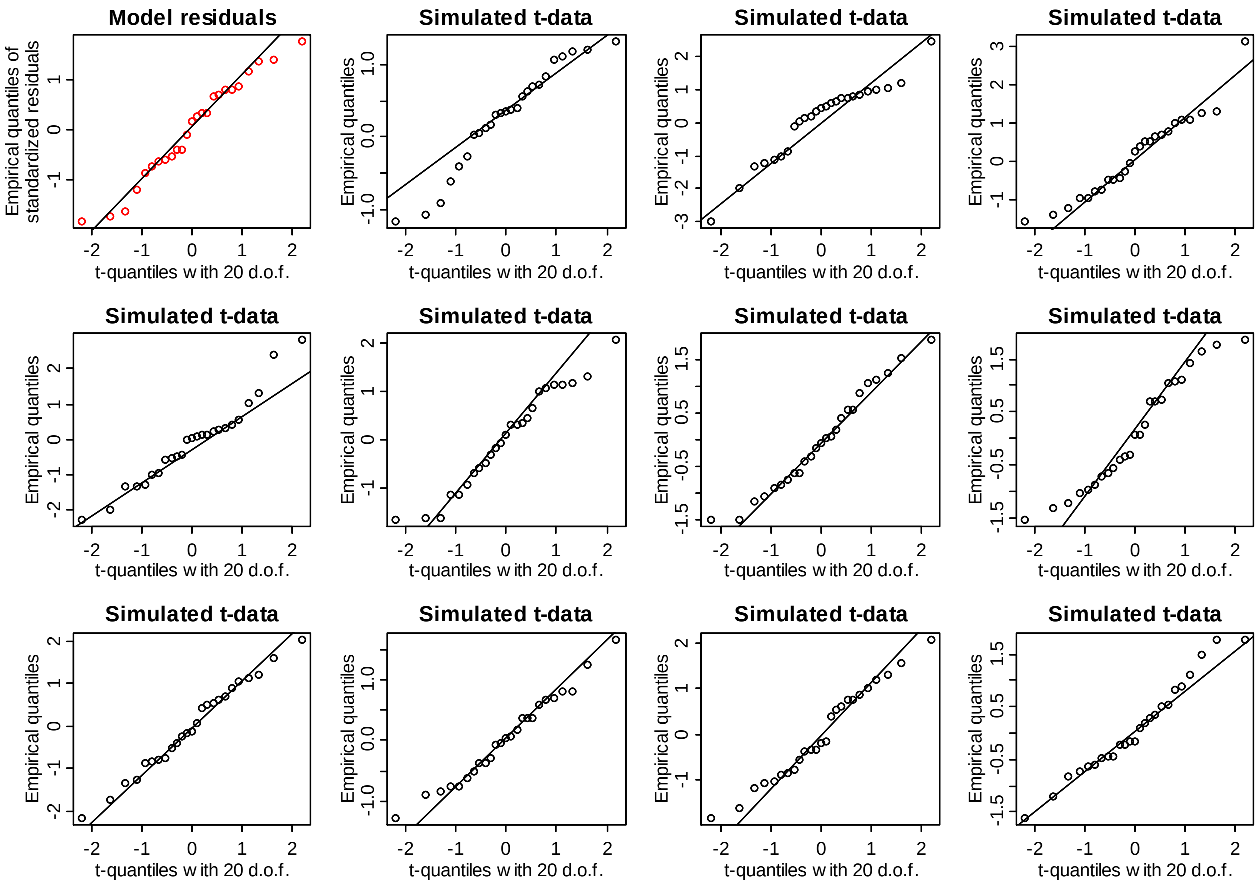

Figure S3 i

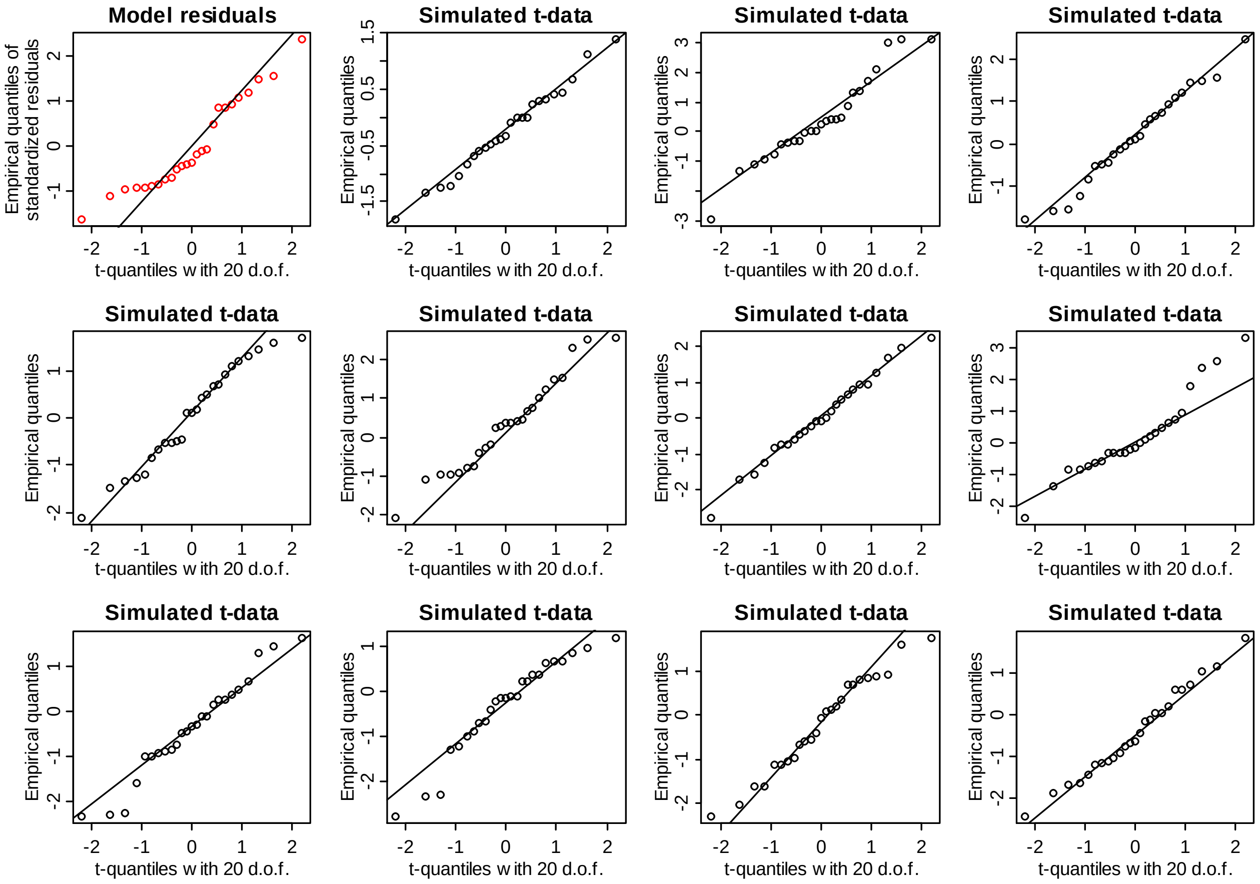

Figure S3 j

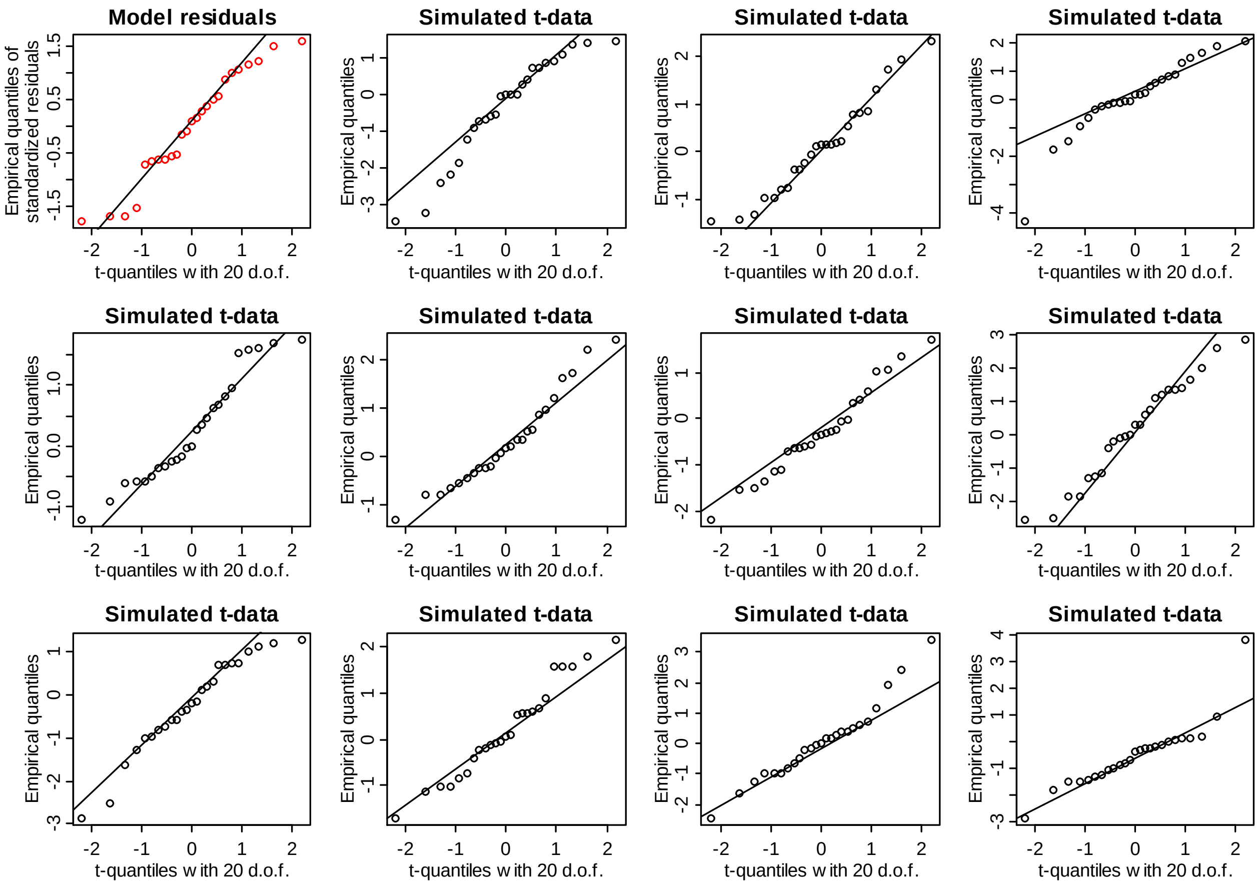

Figure S3 k

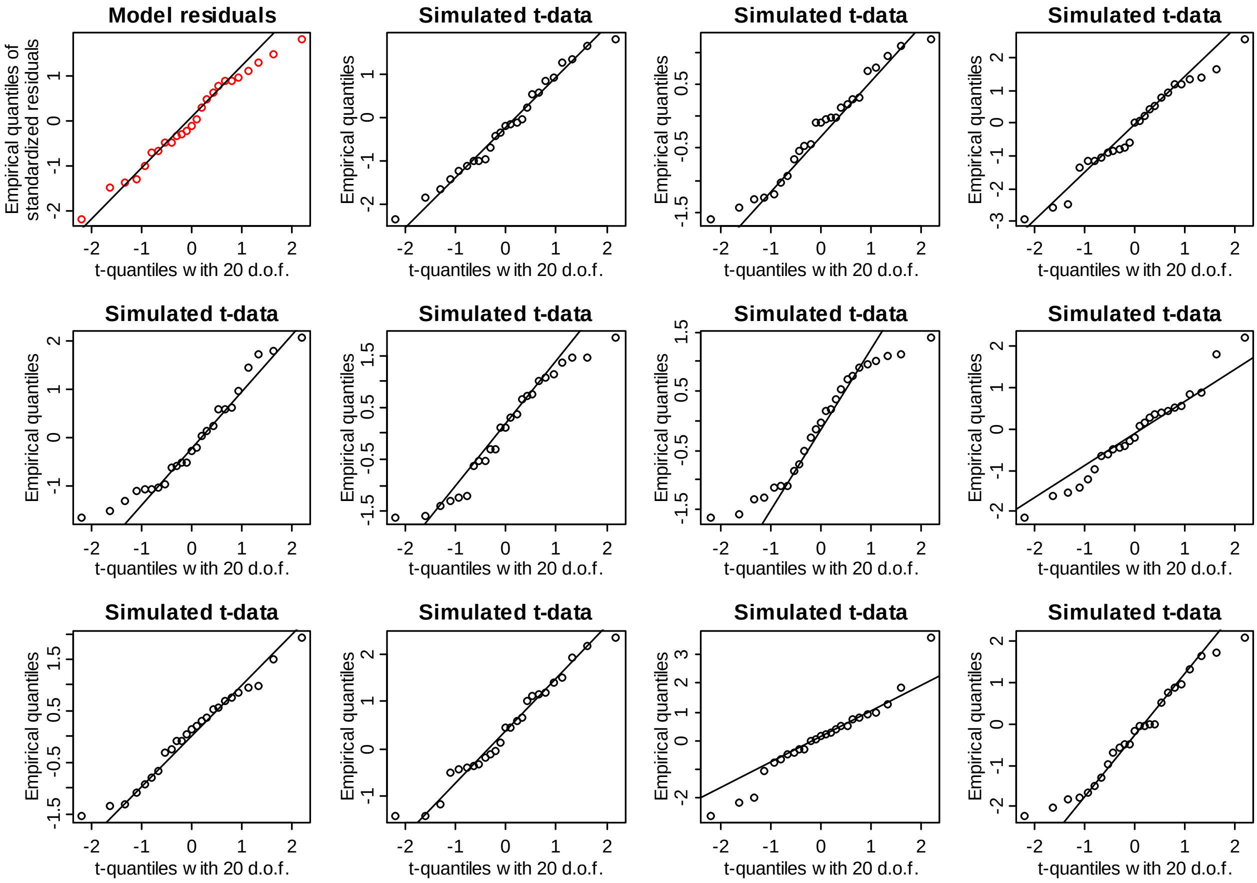

Figure S3 l

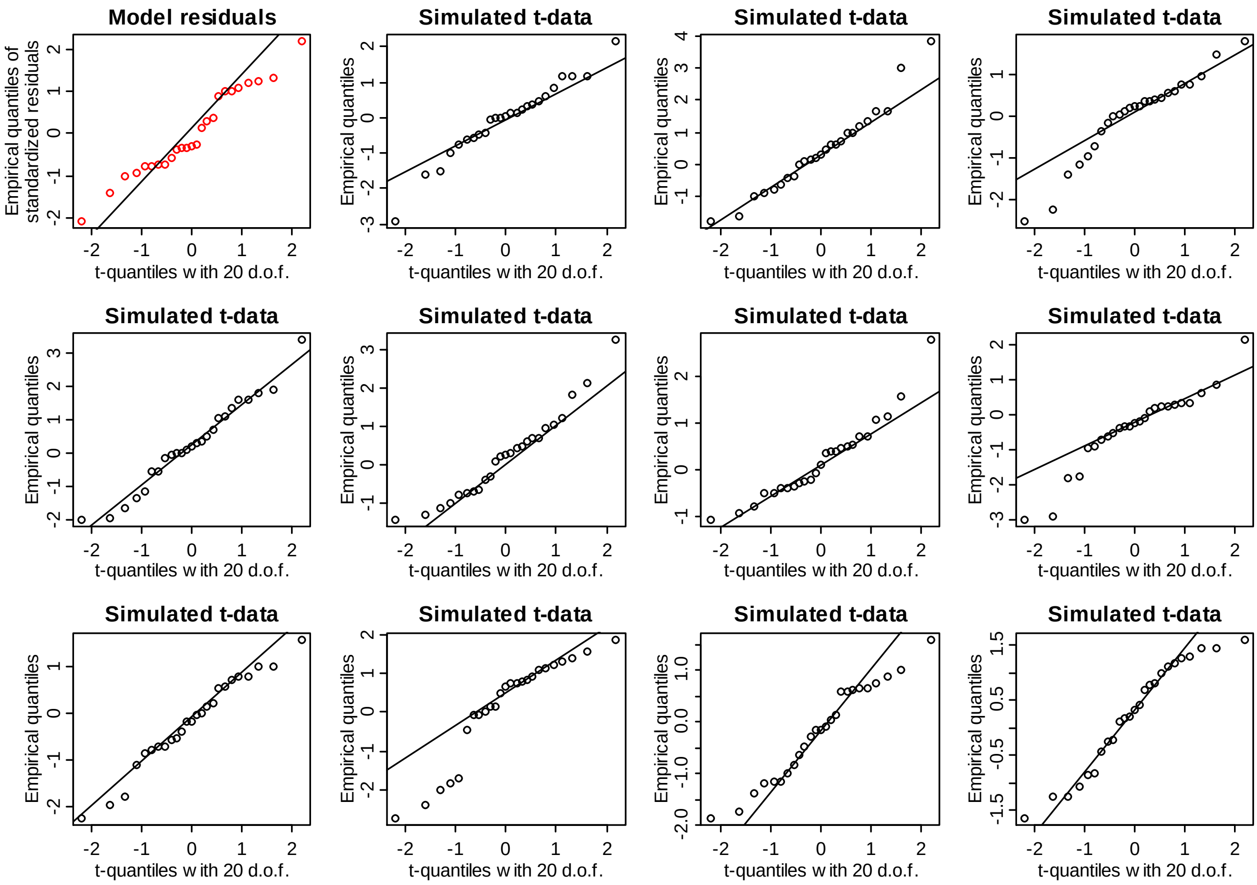

Figure S3 m

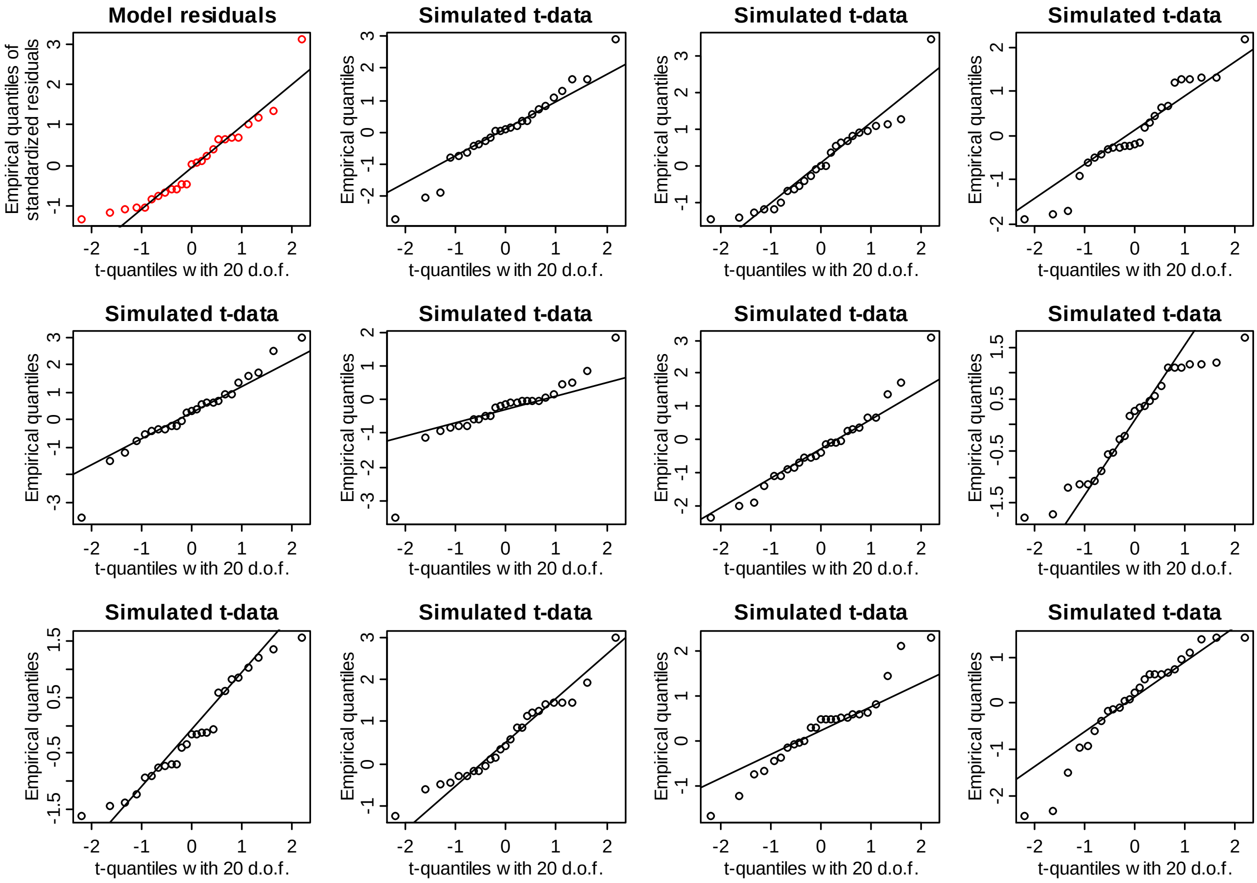

Figure S3 n

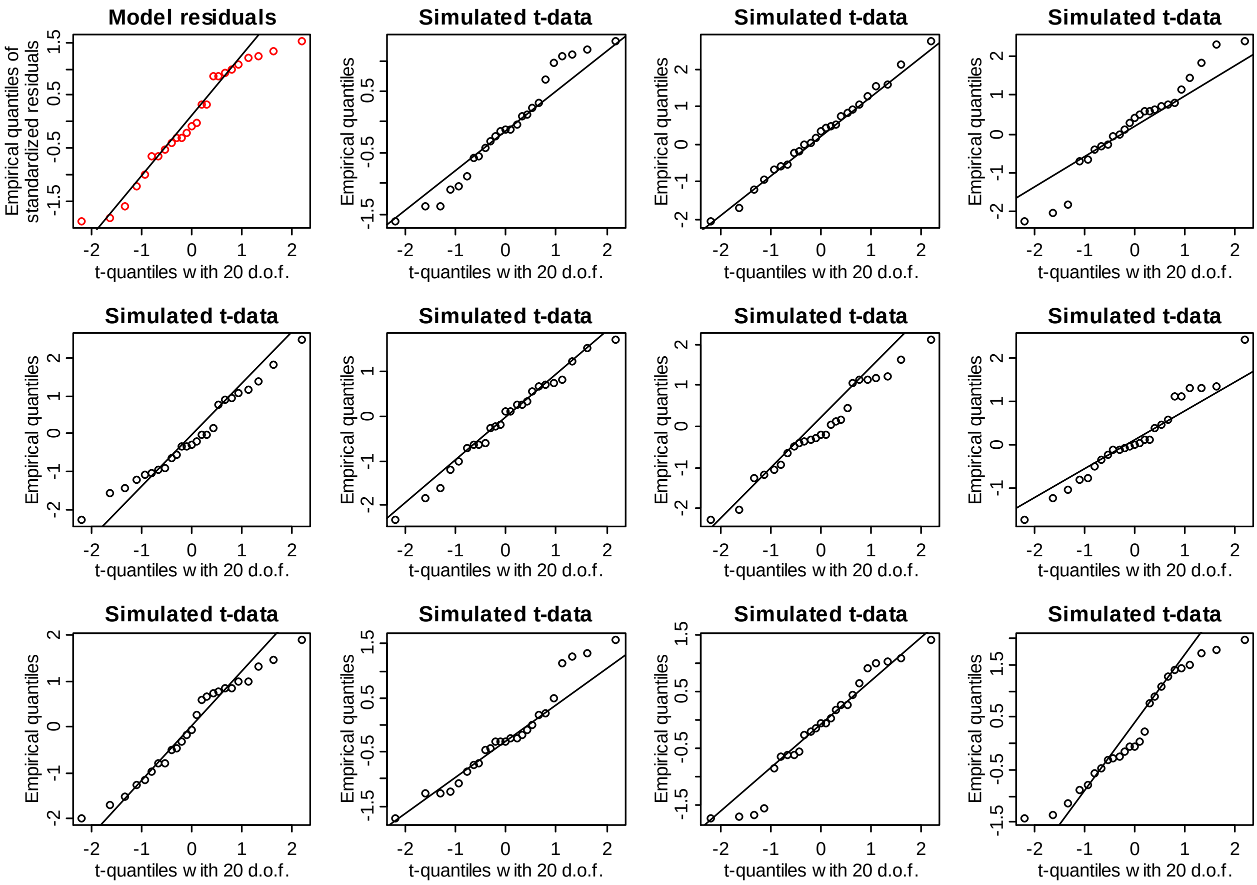

Figure S3 o

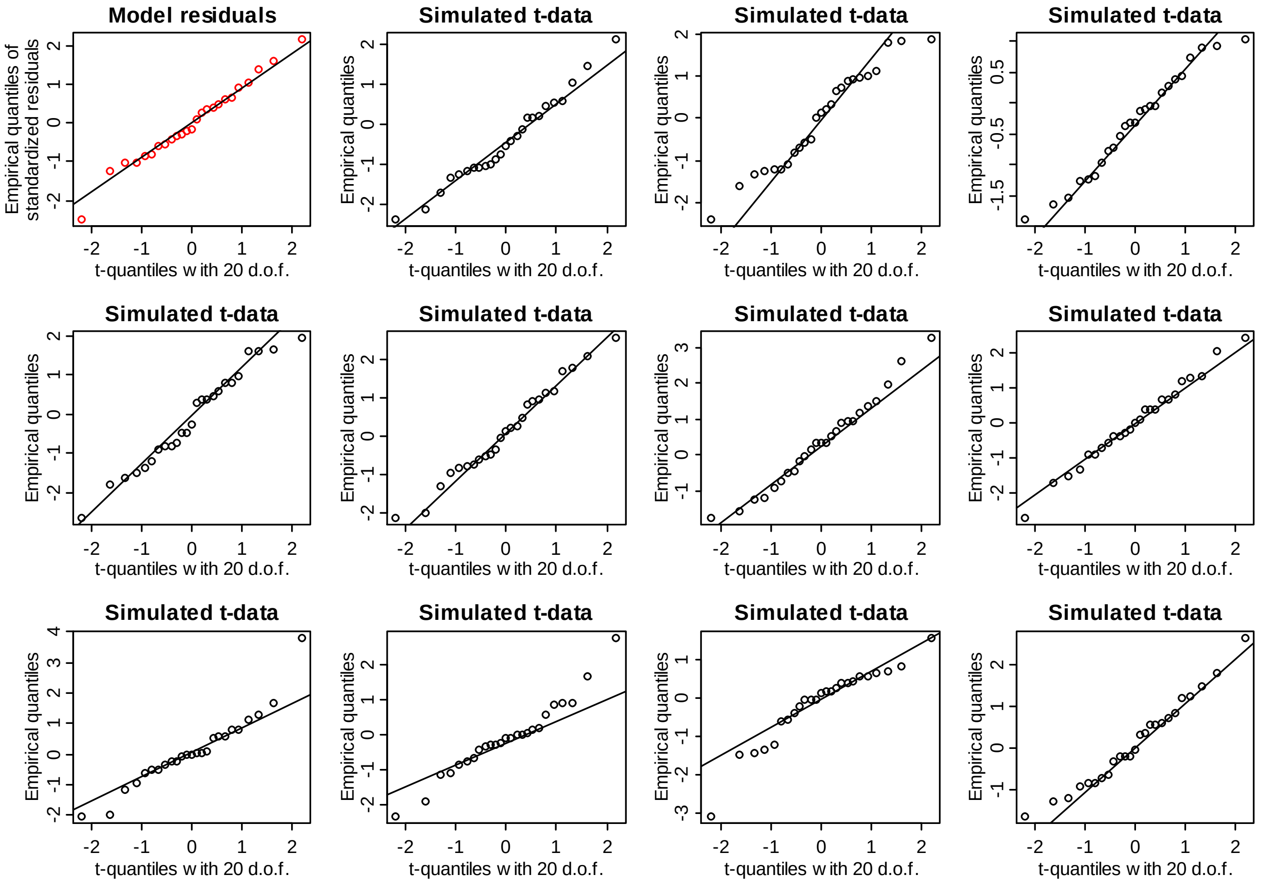

Figure S3 p

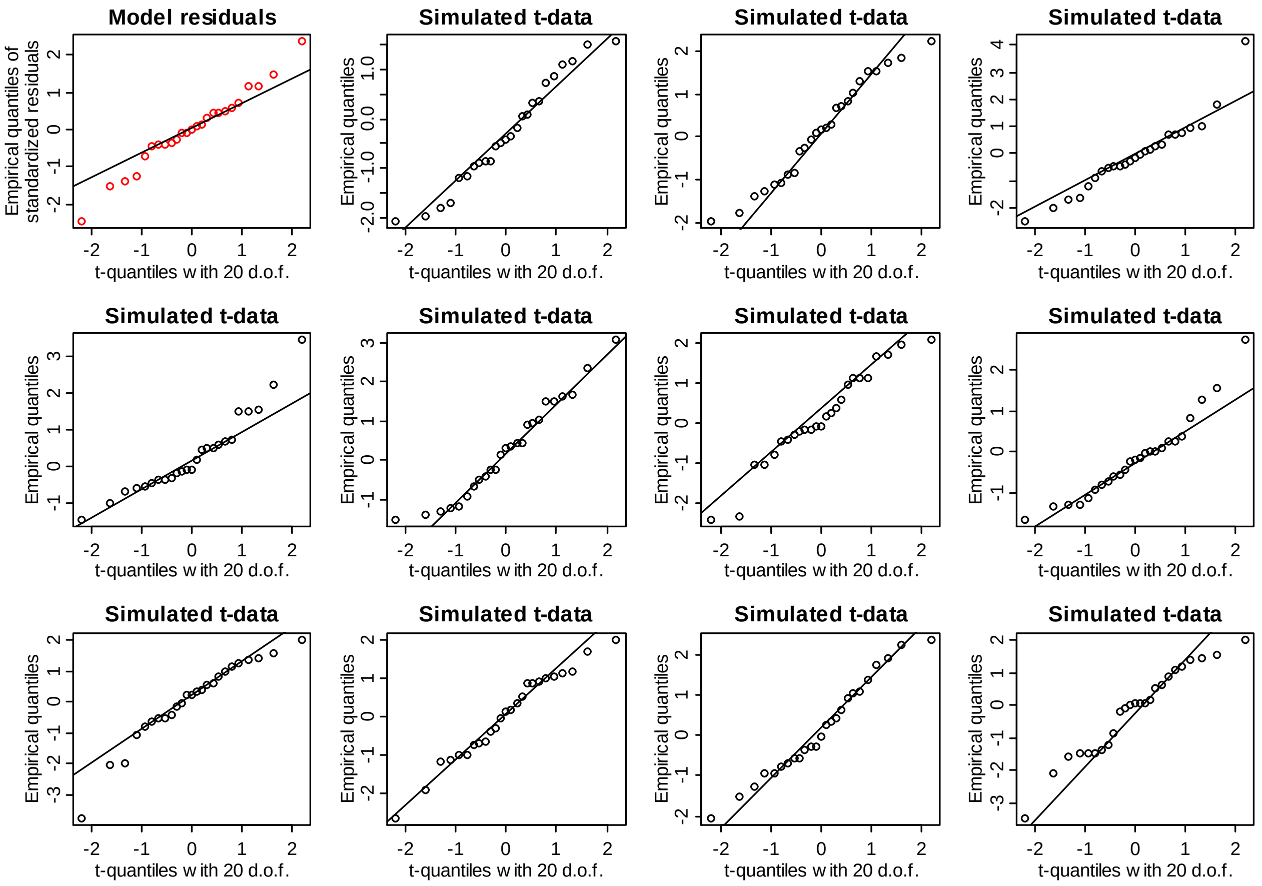

Figure S3 q

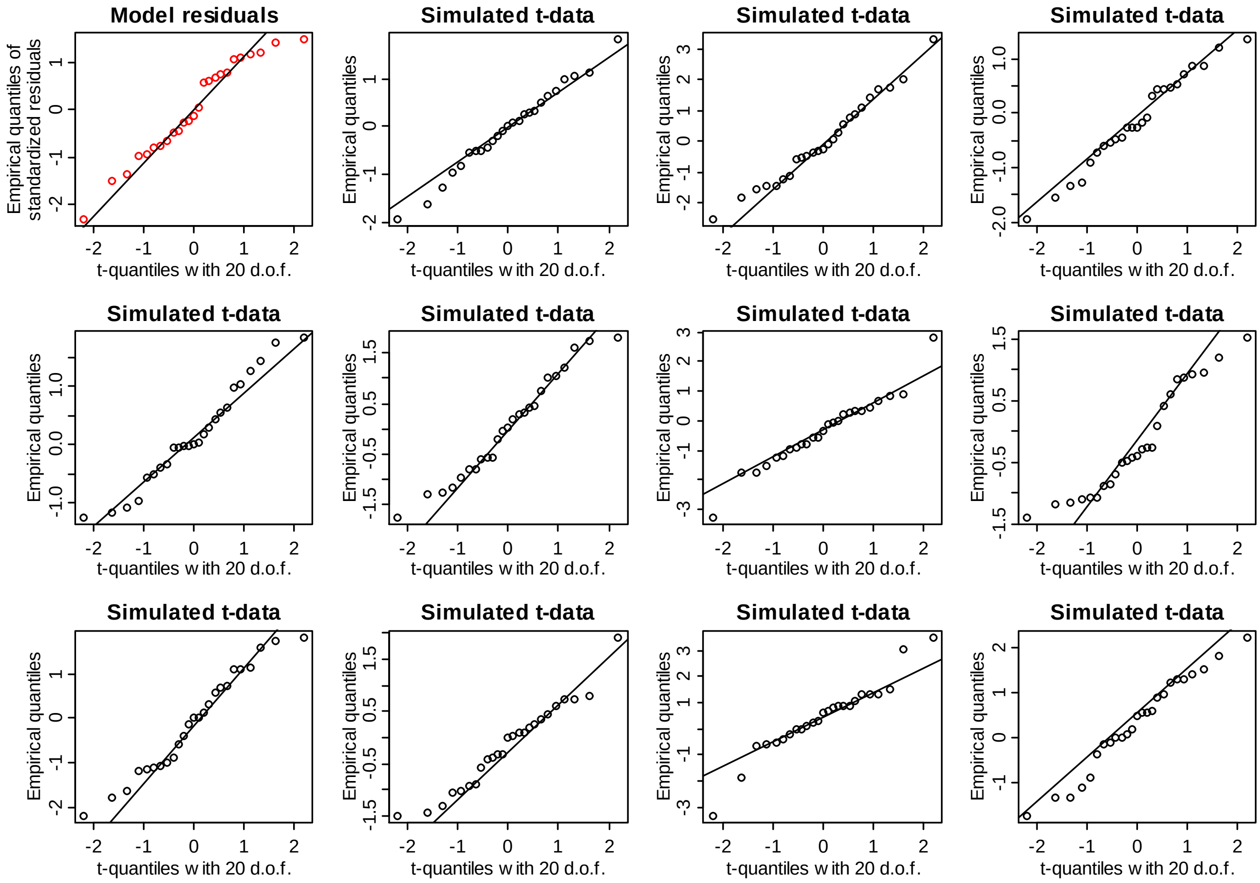

Figure S3 r

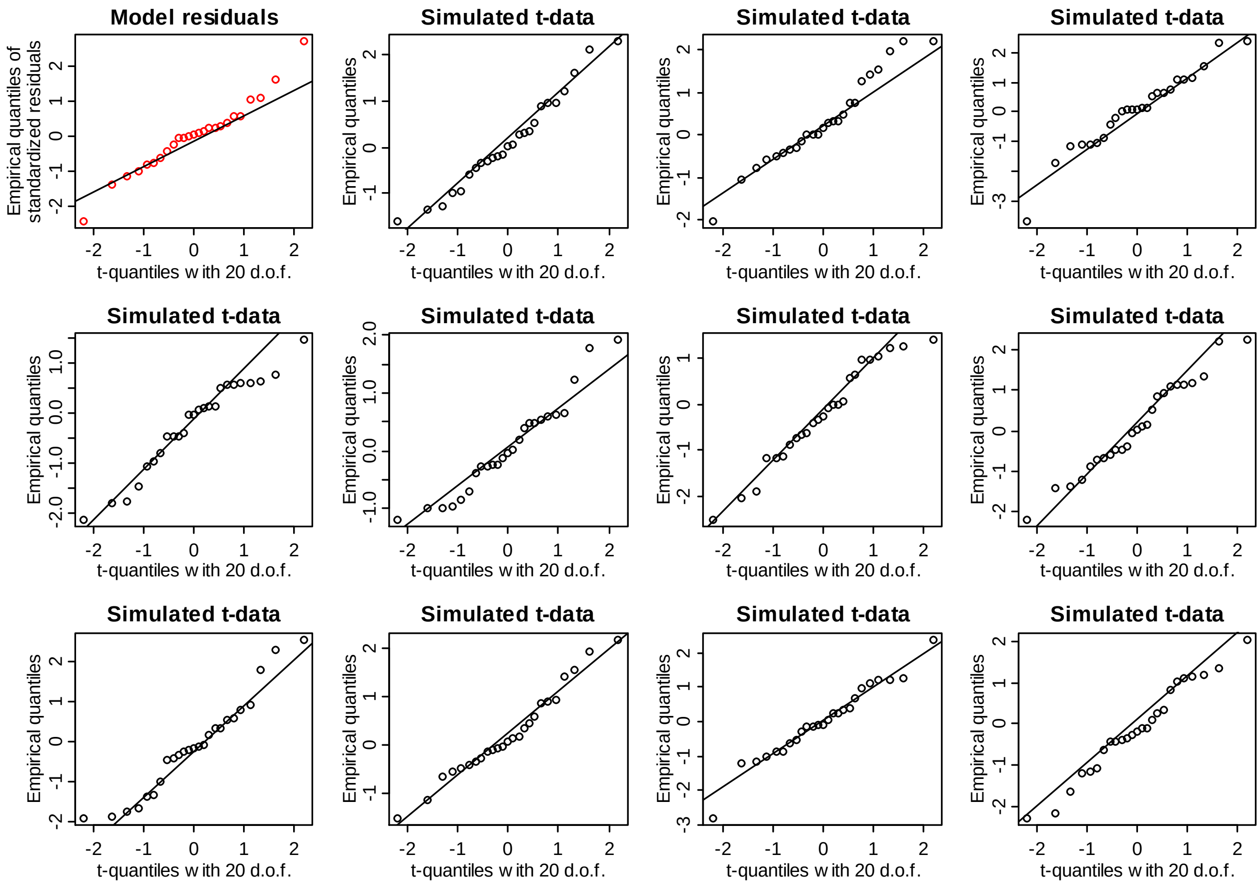

Figure S3 s

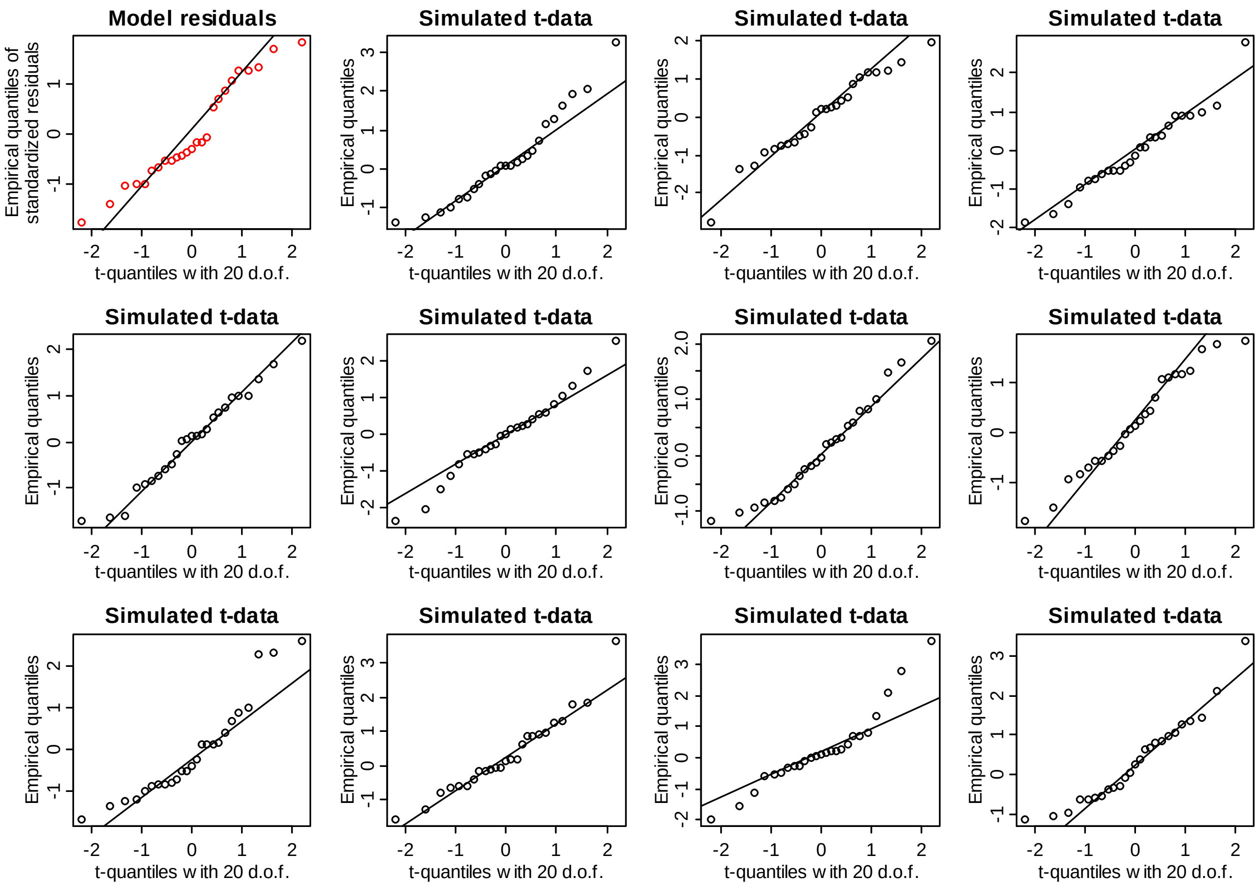

Figure S3 t

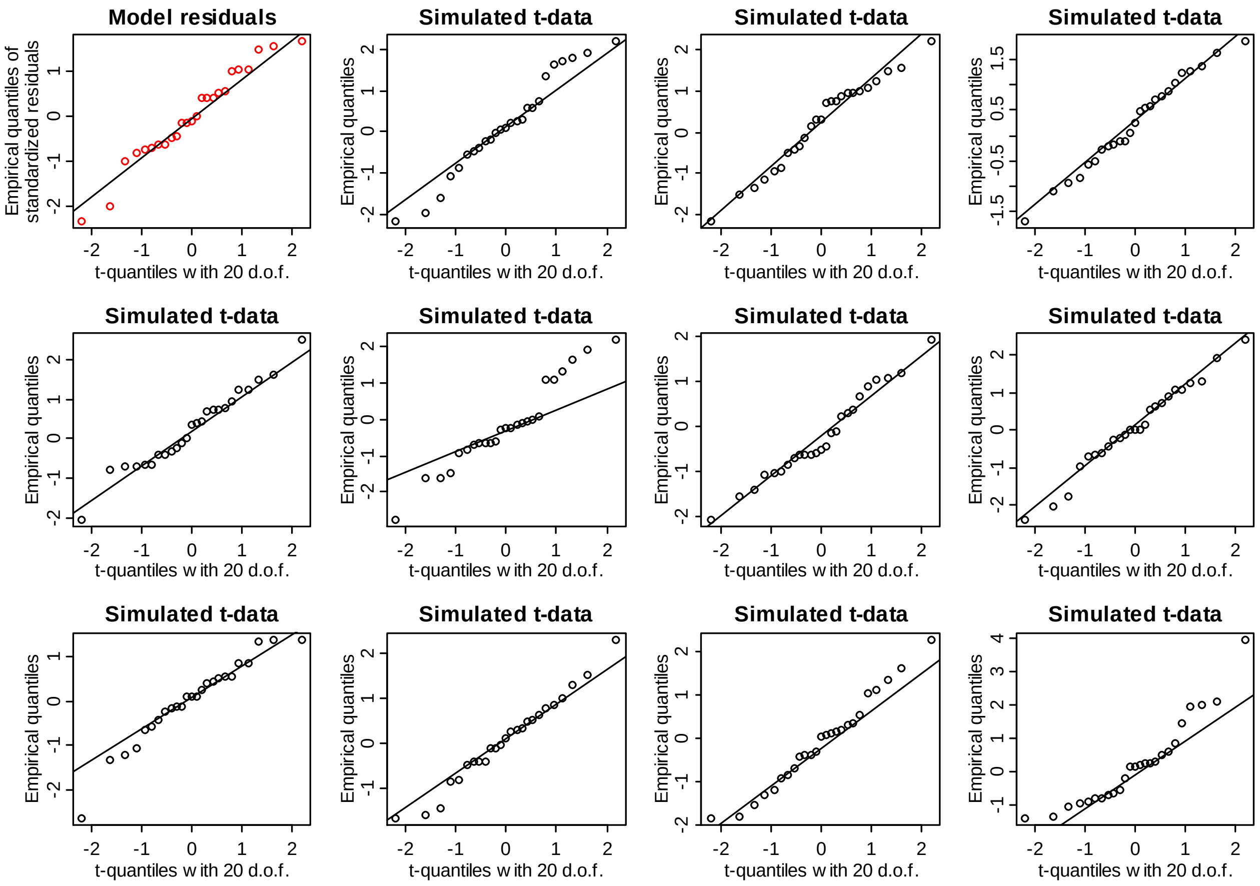

Figure S3 u

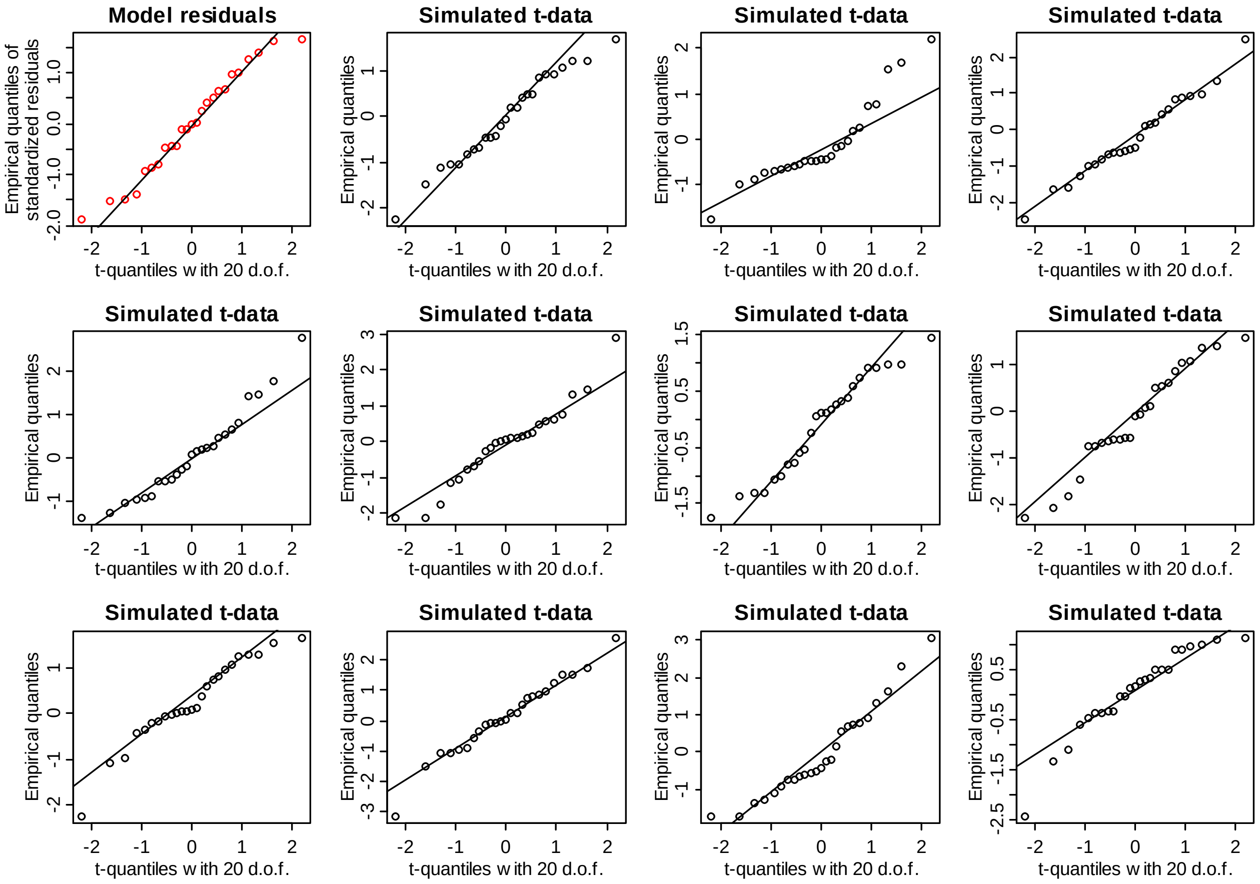

Figure S3 v

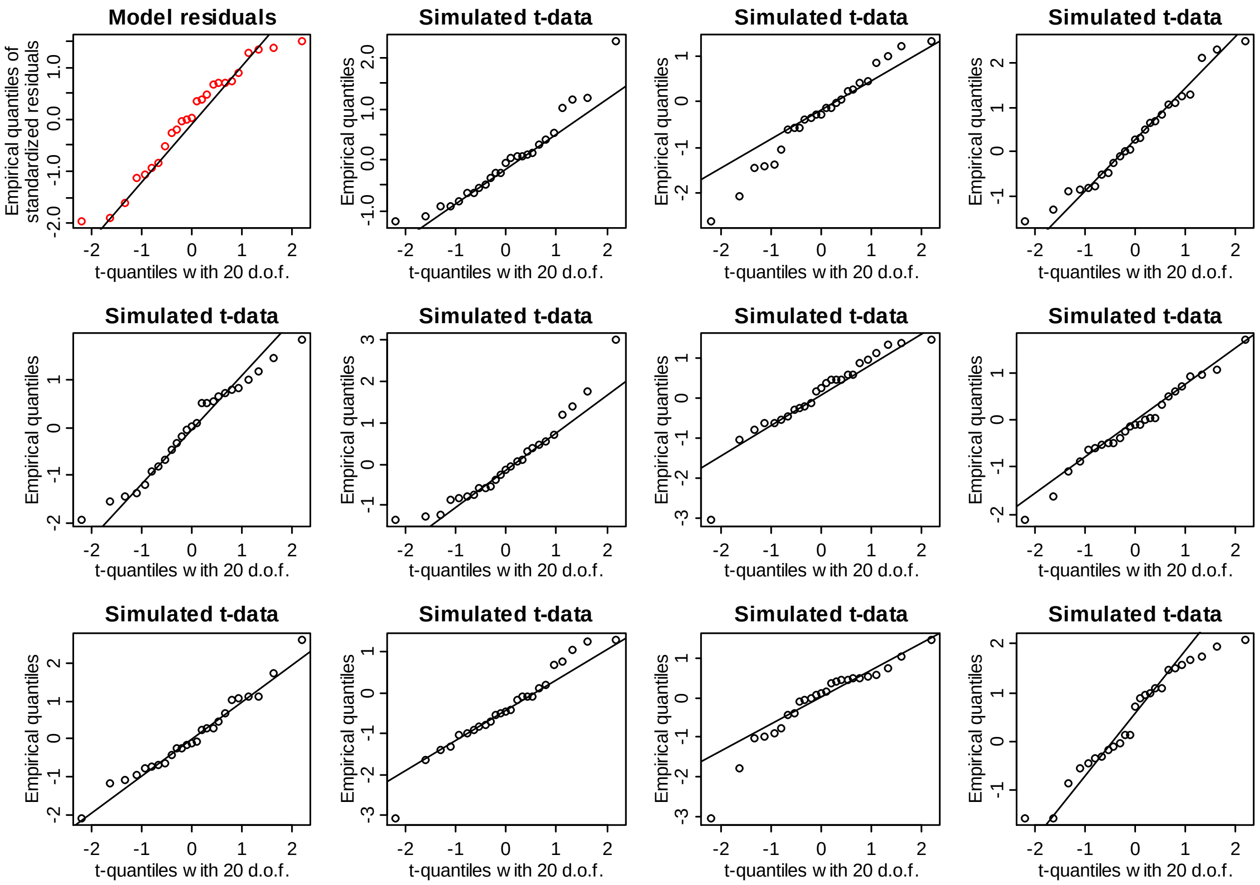

Supplement: Supplementary file 1 — Supplementary information [file 41598_2018_21781_MOESM1_ESM.pdf]
