# Supplementary material for: Population-specific expression of antimicrobial peptides conferring pathogen resistance in the invasive ladybird Harmonia axyridis
Source: Sci Rep. 2018 Feb 26;8:3600. doi: 10.1038/s41598-018-21781-4 (PMC5827023; doi:10.1038/s41598-018-21781-4)
Supplement: Supplementary file 2 — Supplementary File S1 [file 41598_2018_21781_MOESM2_ESM.docx]

**Population-specific expression of antimicrobial peptides conferring pathogen resistance in the invasive ladybird *Harmonia axyridis***

Tobias Gegner^1^, Henrike Schmidtberg^1^, Heiko Vogel^2^ and Andreas Vilcinskas^1,3 *^

^1^ Institute for Insect Biotechnology, Justus-Liebig-University of Giessen, Heinrich-Buff-Ring 26-32, 35392 Giessen, Germany

^2^ Entomology Department, Max-Planck Institute for Chemical Ecology, Hans-Knoell-Strasse 8, 07745 Jena, Germany

^3^ Department of Bioresources, Fraunhofer Institute for Molecular Biology and Applied Ecology, Winchester Strasse 2, 35395 Giessen, Germany

* Corresponding author, email: Andreas.Vilcinskas@agrar.uni-giessen.de

**R script**

library(car)

library(lattice)

library(multcomp)

library(sandwich)

set.seed(20151216)

#input data (can be found as sheet in "File S2")

data <- read.csv("~/R_input_AMPs.csv")

#genewise analysis (run from here until end of script for each gene that was tested by RT-qPCR)

Gen <- "Col1"

D1 <- droplevels(subset(data, subset = gene == Gen))

##one-factoral ANOVA + p-adj with heteroscedasticity consistent estimator "sandwich" for calculating simultanious confidence intervals

fm <- aov(ddct ~ population, data = D1)

fm_glht_sw <- glht(fm, linfct = mcp(population = c("Ger24 - Fr24 = 0","Ger24 - Ko24 = 0","Ger24 - Chi24 = 0","Ger24 - Bio24 = 0",

"Fr24 - Ko24 = 0","Fr24 - Chi24 = 0", "Fr24 - Bio24 = 0",

"Ko24 - Chi24 = 0", "Ko24 - Bio24 = 0",

"Chi24 - Bio24 = 0")), vcov = sandwich)

##statistics - output

out <- file(paste0("AMP-analysis_", Gen, "_statistics.txt"), open = "wt")

sink(out)

sink(out, type = "message")

"summary aov"

summary(fm)

""

""
